# Risk factors for major adverse cardiovascular events after coronary artery bypass grafting using radial artery grafts

**DOI:** 10.3389/fcvm.2023.1238161

**Published:** 2023-09-27

**Authors:** Tianyu Xia, Bo Li, Wei Zhang, Zhe Wang, Xiaofeng Ye, Mi Zhou, Haiqing Li, Jiapei Qiu, Yunpeng Zhu, Qiang Zhao

**Affiliations:** ^1^ Department of Cardiovascular Surgery, Ruijin Hospital, Shanghai Jiao Tong University School of Medicine, Shanghai, China; ^2^ Department of Cardiology, Naval Medical Center, Shanghai, China; ^3^ Department of Biostatistics, School of Public Health, Fudan University, Shanghai, China

**Keywords:** coronary artery disease, coronary artery bypass grafting, radial artery, major adverse cardiovascular events, risk factor

## Abstract

**Background:**

Coronary artery bypass grafting using radial artery grafts (RA-CABG) has improved long-term outcomes. However, major adverse cardiovascular events (MACE-4, including all-cause death, myocardial infarction, stroke, and repeat revascularization) after RA-CABG still occur and the predictors remain uncertain. This study aimed to detect independent risk factors of MACE-4 after RA-CABG.

**Methods:**

This is a retrospective case-control study (NCT04935086) conducted among patients who underwent primary isolated RA-CABG between 2009 and 2019 in our center. Baseline characteristics, procedure characteristics, and medication use were compared to identify the independent predictors of MACE-4, all-cause death, and myocardial infarction (MI) with univariate and then multivariate logistic regression.

**Results:**

A total of 370 patients were analyzed using a mean follow-up duration of 48.8 ± 41.0 months. MACE-4, all-cause death, and MI occurred in 102 (27.6%), 27 (7.3%), and 66 patients (17.8%), respectively. Multivariate analysis revealed prior MI (OR = 2.12, 95%CI 1.05–4.25, *P* = 0.04) and RA to the left anterior descending artery (LAD) (non-left internal mammary artery to LAD) (OR = 4.87, 95%CI 1.41–16.82, *P* = 0.01) as independent predictors of MACE-4 after surgery. Female (OR = 4.53, 95%CI 1.06–19.41, *P* = 0.04), left ventricular ejection fraction (LVEF) <40% (OR = 21.00, 95%CI 1.20–368.35, *P* = 0.04), and RA to LAD (OR = 8.55, 95%CI 1.35–54.10, *P* = 0.02) were independent predictors of all-cause death. Prior MI (OR = 3.11, 95%CI 1.40–6.94, *P* = 0.006) emerged as an independent predictor of MI.

**Conclusion:**

Our data suggested that prior MI and RA to LAD were independent predictors of MACE-4 after RA-CABG. Being female, having an LVEF < 40% and RA to LAD indicated death. Prior MI indicated new MI.

## 1. Introduction

Since being introduced in coronary artery bypass grafting (CABG) in the 1970s, radial artery (RA) is currently the second choice of arterial grafts recommended by American and European guidelines (1–3). Abundant studies have proved the excellent efficacy of CABG using RA (RA-CABG), especially over CABG using saphenous vein graft (SVG) as the second graft (4–11). However, in the RADIAL study, the incidence of major adverse cardiovascular events (MACE-4, including all-cause death, myocardial infarction, stroke, and revascularization) was still appreciable with about 13% at 5 years and 30% at 10 years (8). Unlike risk factors for the prognosis of CABG with SVG being widely investigated, predictors for MACE-4 after RA-CABG remain uncertain (12).

Hence, this retrospective case-control study was conducted among patients who underwent primary isolated RA-CABG in our center over the past decade and aimed to detect independent risk factors for MACE-4 after RA-CABG in baseline characteristics, procedure characteristics, or postoperative medication use.

## 2. Patients and methods

### 2.1. Ethical statement

The case-control study was approved (2020388) by the institutional ethics committee on 16.12.2020, and informed consent was waived. The study was registered with Clinicaltrials.gov (NCT04935086) before enrollment.

### 2.2. Participants

All consecutive adult patients underwent primary isolated RA-CABG in the Department of Cardiovascular Surgery in Ruijin Hospital, Shanghai Jiao Tong University School of Medicine between January 2009 and December 2019. Meanwhile patients with unavailable follow-up records were excluded.

Cases were patients with any MACE-4 occurrence after surgery until the latest follow-up appointment, and controls were the other patients without any MACE-4 occurrence.

### 2.3. Surgical strategy

After administering anesthesia, the median sternotomy is performed to gain access to the heart. Unless there is a risk of hemodynamic instability, all procedures are carried out with the off-pump. The left internal mammary artery (LIMA) is consistently anastomosed to the diseased left anterior descending artery (LAD). However, if the application of LIMA is contraindicated, the right internal mammary artery (RIMA) or RA will be considered an alternative. Both LIMA and RIMA are typically chosen as the pedicled graft provided the risk of inadequate sternal blood supply is low. An Allen test is performed preoperatively to confirm the suitability of the RA. Pedicled RA is preferably utilized to revascularize the second most important diseased coronary, which is based on the preoperative angiography and decided by the operating surgeon. In addition, only the RAs that had not been subjected to catheterization will be considered for procedure. For the remaining coronary targets, skeletonized SVG is commonly harvested and used as a supplement graft. The proximal ends of RA and SVG are anastomosed to the aorta, and the Y/T configuration will be considered if the length of the grafts is limited. The intraoperative assessment of graft patency is conducted using a transit time flowmeter. After ensuring hemostasis and closing the chest, patients are transferred to the Cardiovascular Surgery Intensive Care Unit for postoperative monitoring and care. Additionally, patients will be asked to take an oral calcium channel blocker (CCB) from day 3 and continue this regime for at least 6 months after surgery. After that, it is at the discretion of doctors whether to continue the CCB in patients with hypertension.

### 2.4. Outcomes and potential predictors

The definitions of MACE-4 components followed definitions of the consensus report published jointly by ACCF and AHA in 2017 (13). In this study, MACE-4 was defined as a composite of all-cause death, myocardial infarction [MI], stroke, and repeat revascularization. The definition of MACE-3 was, however, a composite of cardiovascular death (CV-death), MI, and stroke.

Potential predictors for MACE-4 after RA-CABG included baseline characteristics, procedure characteristics, and medication use after surgery. Some definitions of candidate risk factors are listed in the supplement.

### 2.5. Data collection

A follow-up database was established through outpatient visits or via telephone by cardiac surgeons as a part of standard institutional procedures. During follow-up, if any component of MACE-4 was reported, patients or family members were asked to elaborate on the details, including the date, specific type, and related medical records for confirmation. If an accurate date of occurrence was unable to be acquired, it would be estimated as the median of a limited time window, outside of which MACE-4 did not occur with affirmation.

The baseline data were extracted from electronic medical records, and incomplete electronic medical records were supplemented with paper documentation stored in the Medical Record Department.

### 2.6. Bias

Baseline database was built by two researchers separately, and differences were revised by a third researcher to guarantee accuracy and completeness. De-identification was also used during the whole data collection process to ensure an objective database.

### 2.7. Study size

Even with an estimated 20% occurrence of MACE-4, the power of the analysis was limited to 370 patients. The power to detect potential associations depended on the prevalence of risk factors among both cases and controls, the magnitude of the risk conferred, and the incidence of MACE-4. The analyses had 80% power to detect an OR of 2.0 for risk factors with a prevalence of 15% or greater at a 5% (two-tailed) significance level or an OR of 2.5 for risk factors with a prevalence of 6% or greater.

### 2.8. Statistical analysis

Apart from MACE-4, risk factors for MACE-3, all-cause death, CV-death, MI, stroke, and repeat revascularization were investigated as well. Risk factors for all clinical outcomes in the perioperative period (day 0 to day 30) and the early period (day 31 to 3 years) were also studied separately.

Continuous variables were presented as the mean ± standard deviation (SD) or median (IQR), while categorical variables were presented as frequency (percentages). Logistic regression was used to explore the risk factors of clinical outcomes of interest. The factors with a *P*-value ≤0.10 in the univariate logistic analysis were subsequently included in the multivariate logistic regression. ORs were shown with 95% CIs. A *P*-value <0.05 was considered statistically significant. As for the missing baseline data of patients with available follow-up records, analyses were only performed after the exclusion of those patients with missing baseline data. All statistical analyses were performed with SAS software (version 9.4).

## 3. Results

### 3.1. Participants

There were 385 primary isolated RA-CABG patients during the defined period. After accessing clinical research database, 370 patients (96.1%) with available follow-up records were included (see Supplementary Figure S1).

### 3.2. Descriptive data

Table 1 summarized selected baseline characteristics of the 370 patients. But, even complemented with paper medical documentation, there are still a few items with missing data. In total, 27 patients without MACE occurrence and 13 patients with MACE occurrence have incomplete baseline data.

**Table 1:** Selected baseline characteristics of patients in the present study^a^.

	MACE-4		*P*-value
	Yes (N1 = 102)	No (N2 = 268)	
	*n* (%)	*n* (%)	
Demographics			
Age (years)	56.0 ± 10.3	54.4 ± 9.4	.17
Female	10 (9.8)	24 (9.0)	.80
Body mass index (kg/m^2^, mean ± SD)	25.3 ± 3.3	25.3 ± 2.9	.92
Medical history			
Hypertension	80 (78.4)	200 (74.6)	.45
Diabetes mellitus	37 (36.3)	100 (37.3)	.85
Dyslipidemia	45 (44.6)	138 (52.5)	.18
CKD	10 (9.8)	18 (6.7)	.67
PVD	7 (8.2)	18 (8.3)	>.99
COPD	4 (5.3)	5 (2.6)	.46
Smoking history	69 (67.6)	167 (62.3)	.34
Coronary artery lesions			
LM stenosis ≥ 50%	26 (28.0)	83 (33.9)	.30
LM disease only	2 (2.2)	6 (2.4)	.81
Single-system disease	4 (4.3)	15 (6.1)	.52
Two-system disease	21 (22.6)	64 (26.1)	.50
Three-system disease	66 (71.0)	160 (65.3)	.32
Clinical presentation			
Stable angina	23 (22.5)	64 (23.9)	.79
Unstable angina	56 (54.9)	172 (64.2)	.10
NSTEMI	9 (8.8)	16 (6.0)	.33
STEMI	7 (6.9)	5 (1.9)	.04
LVEF			
≥50%	85 (84.2)	248 (92.5)	.02
40%–49%	12 (11.9)	16 (6.0)	.06
<40%	4 (4.0)	4 (1.5)	.16
NYHA classification			
I	2 (2.0)	7 (2.7)	.97
II	60 (60.6)	168 (63.6)	.59
III	36 (36.4)	89 (33.7)	.64
IV	1 (1.0)	0	.61
Surgical characteristics			
Emergency or urgent surgery	4 (3.9)	19 (7.1)	.26
Elective surgery	70 (68.6)	193 (72.0)	.52
Delayed surgery	28 (27.5)	56 (20.9)	.18
LIMA-LAD	86 (87.8)	242 (96.4)	.002
Off-pump	96 (94.1)	266 (99.3)	.01
TAR	57 (55.9)	168 (62.7)	.23
Complete revascularization	74 (73.3)	209 (79.5)	.20
Distal anastomoses (mean ± SD)	3.2 ± 0.9	3.1 ± 1.0	.35
Arterial distal anastomoses (mean ± SD)	2.5 ± 0.8	2.6 ± 0.7	.76
Venous distal anastomoses (mean ± SD)	0.7 ± 0.8	0.5 ± 0.8	.16

CKD, chronic kidney disease; COPD, chronic obstructive pulmonary disease; LAD, left anterior descending artery; LIMA, left internal mammary artery; LM, left main coronary artery; LVEF, left ventricular ejection fraction; MI, myocardial infarction; NSTEMI, non-ST segment elevation myocardial infarction; NYHA, New York Heart Association; PVD, peripheral vascular disease; STEMI, ST segment elevation myocardial infarction; TAR, total arterial revascularization.

^a^ N1 and N2 are denominators for groups patients with and without MACE-4; denominators are not always 102 and 268, because some variables were missing for a small number of patients.

### 3.3. Outcome data

**Figure 1:**
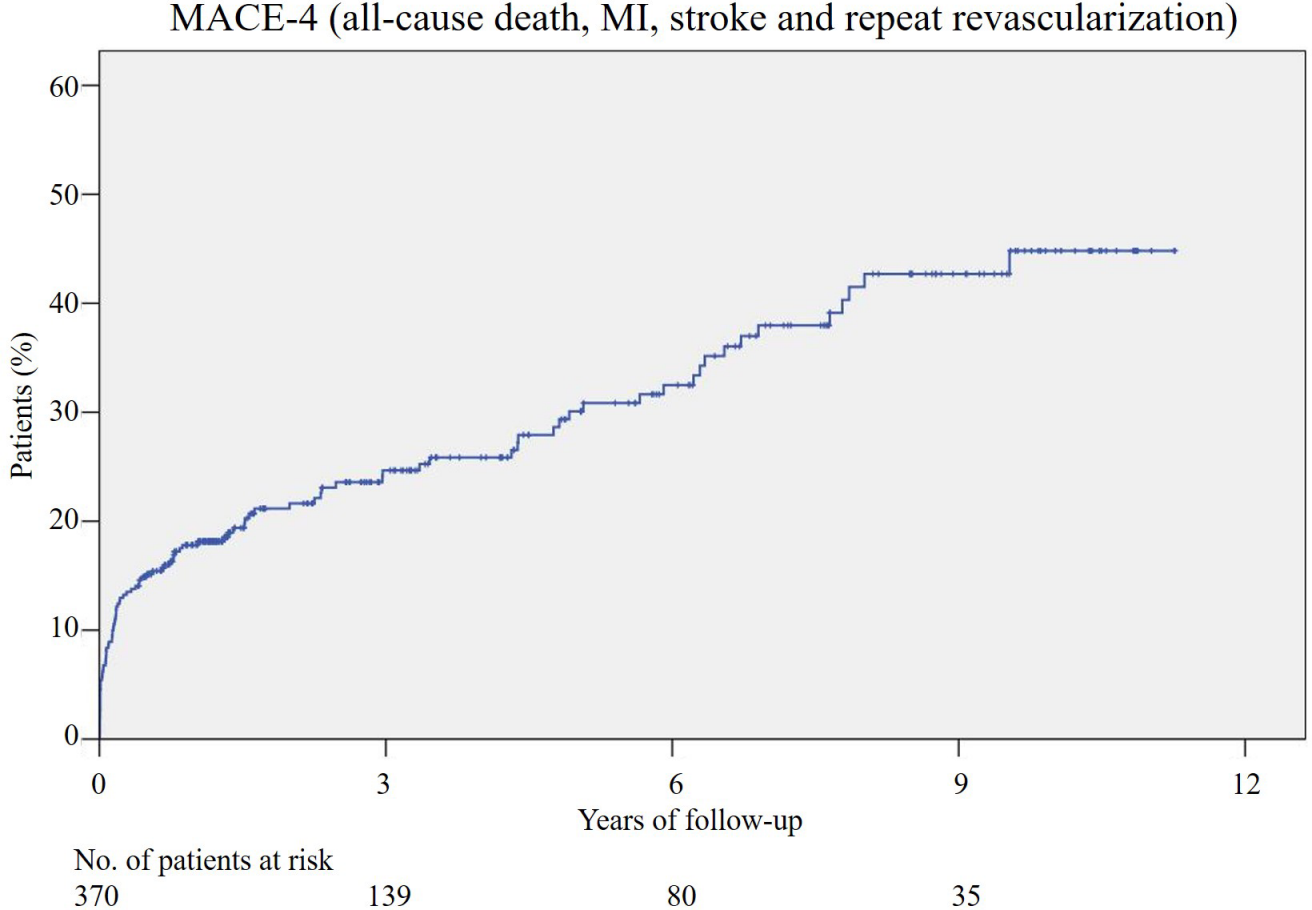
Cumulative incidence of the MACE-4 after RA-CABG. MACE, major adverse cardiovascular events; MI, myocardial infarction; RA-CABG, coronary artery bypass grafting using radial artery.

Ranging from 4 to 137 months, the median duration of follow-up was 34.7 months, and the mean was 48.8 months. During the follow-up, MACE-4 occurred in 102 patients (27.6%) (Figure 1), and MACE-3 occurred in 86 (23.2%) patients. In total, 27 patients (7.3%) died after RA-CABG, and 18 (4.9%) of them were CV-deaths. MI occurred in 66 patients (17.8%) and stroke in 12 (3.2%). Repeat revascularizations completed by percutaneous coronary intervention were conducted in a total of 12 patients (3.2%).

### 3.4. Main results

#### 3.4.1. Predictors for MACE-4

Univariate analysis revealed that an age of 70 or above at admission, MI at admission, pro-BNP > 600 pg/ml, LVESD > 40 mm, LVEDD > 60 mm, LVEF < 50%, prior MI, and a current smoking habit were risk factors of MACE-4. Regarding surgical factors, RA to LAD (non-LIMA to LAD) and on-pump were risk factors (Table 2). The risk factors above were included in multivariate logistic regression (see Supplementary Table S1). After excluding the variable without astringency (on-pump) from multivariate logistic regression modeling, prior MI (OR = 2.12, 95% CI 1.05–4.25, *P* = .05) and RA to LAD (OR = 4.87, 95% CI 1.41–16.82, *P* = .01) were identified to be independently associated with MACE-4 (Table 2).

**Table 2:** Univariate and adjusted multivariate logistic regression analysis of risk factors for MACE-4^a^.

	Univariate estimates			Adjusted estimates		
	OR	95% CI	*P*	OR	95% CI	*P*
Preoperative factors						
Female	1.11	0.51–2.40	.80			
BMI ≥ 24	0.87	0.54–1.39	.55			
BMI ≥ 28	1.40	0.79–2.48	.25			
Aged 60 years or more	1.20	0.75–1.94	.45			
Aged 65 years or more	1.30	0.72–2.38	.39			
Aged 70 years or more	2.62	1.15–5.94	.02	2.16	0.71–6.63	.18
ACS at admission	1.03	0.63–1.69	.90			
MI at admission	2.35	1.19–4.67	.01	1.52	0.63–3.68	.35
Diabetes mellitus	0.96	0.60–1.54	.85			
Hypertension	1.24	0.72–2.13	.45			
COPD	2.12	0.55–8.13	.27			
Hyperlipemia	0.73	0.46–1.15	.18			
CKD	1.51	0.67–3.39	.32			
PVD	1.00	0.40–2.48	>.99			
Prior MI	2.69	1.68–4.30	<.001	2.12	1.05–4.25	.04
Prior stroke	1.13	0.53–2.37	.76			
Smoking history	1.26	0.78–2.05	.34			
Current smoking	1.59	1.01–2.53	.05	1.37	0.74–2.54	.31
NYHA classification III or IV	0.87	0.54–1.41	.56			
Pro-BNP > 600pg/ml	2.28	1.13–4.61	.02	1.17	0.47–2.94	.73
LVESD > 40mm	3.92	1.87–8.22	.001	3.32	0.77–14.25	.11
LVEDD > 60mm	3.20	1.13–9.05	.03	0.71	0.14–3.66	.68
LVEF < 40%	2.72	0.67–11.09	.16			
LVEF < 50%	2.34	1.16–4.71	.02	0.68	0.16–2.87	.60
Anemia	1.34	0.80–2.26	.27			
Abnormal platelet count	1.23	0.58–2.61	.59			
LM stenosis	0.76	0.45–1.28	.30			
Three-system disease	1.30	0.77–2.18	.32			
Surgical factors						
Non-elective operation	1.17	0.71–1.92	.55			
RA to LAD (non-LIMA to LAD)	3.75	1.53–9.22	.004	4.87	1.41–16.82	.01
Non-BIMA	2.33	0.52–10.49	0.27			
Stenosis of RA targeted coronary artery < 70%	2.13	0.77–5.88	0.14			
Arterial grafts < 50%	2.70	0.66–11.03	0.17			
Non-TAR	1.33	0.83–2.13	0.23			
Incomplete revascularization	1.41	0.83–2.38	0.20			
On-pump	8.31	1.65–41.88	0.01			
Postoperative medication						
CCB for 6 months	1.06	0.53–2.15	0.86			

ACS, acute coronary syndrome; BIMA, bilateral internal mammary arteries; BMI, body mass index; CCB, calcium channel blockers; CKD, chronic kidney disease; COPD, chronic obstructive pulmonary disease; LAD, left anterior descending artery; LIMA, left internal mammary artery; LM, left main coronary artery; LVEDD, left ventricular end diastolic diameter; LVEF, left ventricular ejection fraction; LVESD, left ventricular end systolic diameter; MI, myocardial infarction; NYHA, New York Heart Association; Pro-BNP, pro-B-type natriuretic peptide; PVD, peripheral vascular disease; RA, radial artery; TAR, total arterial revascularization.

^a^ Multivariable analysis was conducted again after excluding the variable without astringency (on-pump) from the previous multivariate logistic regression modeling.

#### 3.4.2. Predictors for other outcomes

Univariate and multivariate analyses of independent risk factors for secondary outcomes were listed in Table 3. Prior MI (OR = 3.10, 95% CI 1.41–6.83, *P* = .005) was independently associated with MACE-3 (Table 3, see Supplementary Table S2); female (OR = 4.53, 95% CI 1.06–19.41, *P* = .04), LVEF < 40% (OR = 21.00, 95% CI 1.20–368.35, *P* = .04), and RA to LAD (OR = 8.55, 95% CI 1.35–54.10, *P* = .02) with all-cause death (Table 3, see Supplementary Tables S3, S4); Female (OR = 8.28, 95% CI 1.06–64.62, *P* = .004) and on-pump with CV-death (Table 3, see Supplementary Table S5); Prior MI (OR = 3.11, 95% CI 1.40–6.94, *P* = .006) with postoperative MI (Table 3, see Supplementary Table S6); there was no independent risk factor associated with stroke (see Supplementary Table S7); current smoking (OR = 5.95, 95% CI 1.28–27.75, *P* = .02) was associated with repeat revascularization (Table 3, see Supplementary Table S8).

**Table 3:** Independent risk factors for secondary outcomes identified by univariate and multivariate analysis^a^.

	Univariate estimates			Adjusted estimates		
	OR	95% CI	*P*	OR	95% CI	*P*
MACE-3						
Prior MI	3.48	2.10–5.78	<.001	3.10	1.41–6.83	.005
All-cause death^b^						
Female	3.21	1.20–8.62	.02	4.53	1.06–19.41	.04
LVEF < 40%	15.41	3.61–65.79	<.001	21.00	1.20–368.35	.04
RA to LAD (non-LIMA to LAD)	3.44	1.06–11.14	.04	8.55	1.35–54.10	.02
CV-death						
Female	3.07	0.95–9.91	.06	8.82	1.06–64.62	.04
On-pump	24.85	5.63–109.76	<.001	105.6	3.66–3,044.7	.007
MI						
Prior MI	3.78	2.17–6.58	<.001	3.11	1.40–6.94	.006
Repeat revascularization						
Current smoking	5.35	1.16–24.75	.03	5.95	1.28–27.75	.02

CV-death, cardiovascular death; LAD, left anterior descending artery; LIMA, left internal mammary artery; LVEF, left ventricular ejection fraction; MACE, major adverse cardiovascular events; MI, myocardial infarction; RA, radial artery.

^a^ No independent risk factor for stroke was found; only univariate and multivariate analyses of independent risk factors were listed above.

^b^ Multivariable analysis for all-cause death was conducted again after excluding the variables without astringency.

#### 3.4.3. Additional analyses

In the perioperative period, MI at admission was independently associated with both MACE-4 and MI, but no independent risk factors for other outcomes were identified (see Supplementary Tables S9–S15). As for the early period, prior MI emerged as an independent predictor for MACE-3 (see Supplementary Table S16); chronic kidney disease (CKD) and on-pump for all-cause death (see Supplementary Table S17); prior MI for postoperative MI (see Supplementary Table S18). However, no independent predictor for MACE-4, CV-death, stroke, and repeat revascularization was found (see Supplementary Tables S19–S22).

### 3.5. Sensitivity analysis

Although revealed as potential independent risk factors (*P* ≤ 0.1) of MACE-4 or all-cause death by univariate analyses, several variables were found to be without astringency after being included in multivariate analyses (see Supplementary Tables S1, S3). So multivariate analyses were applied again after the exclusion of these variables. Finally, independent risk factors of MACE-4 were unchanged; but in addition to being female, LVEF < 40% and RA to LAD emerged as independent risk factors of all-cause death as well.

## 4. Discussion

**Figure 2:**
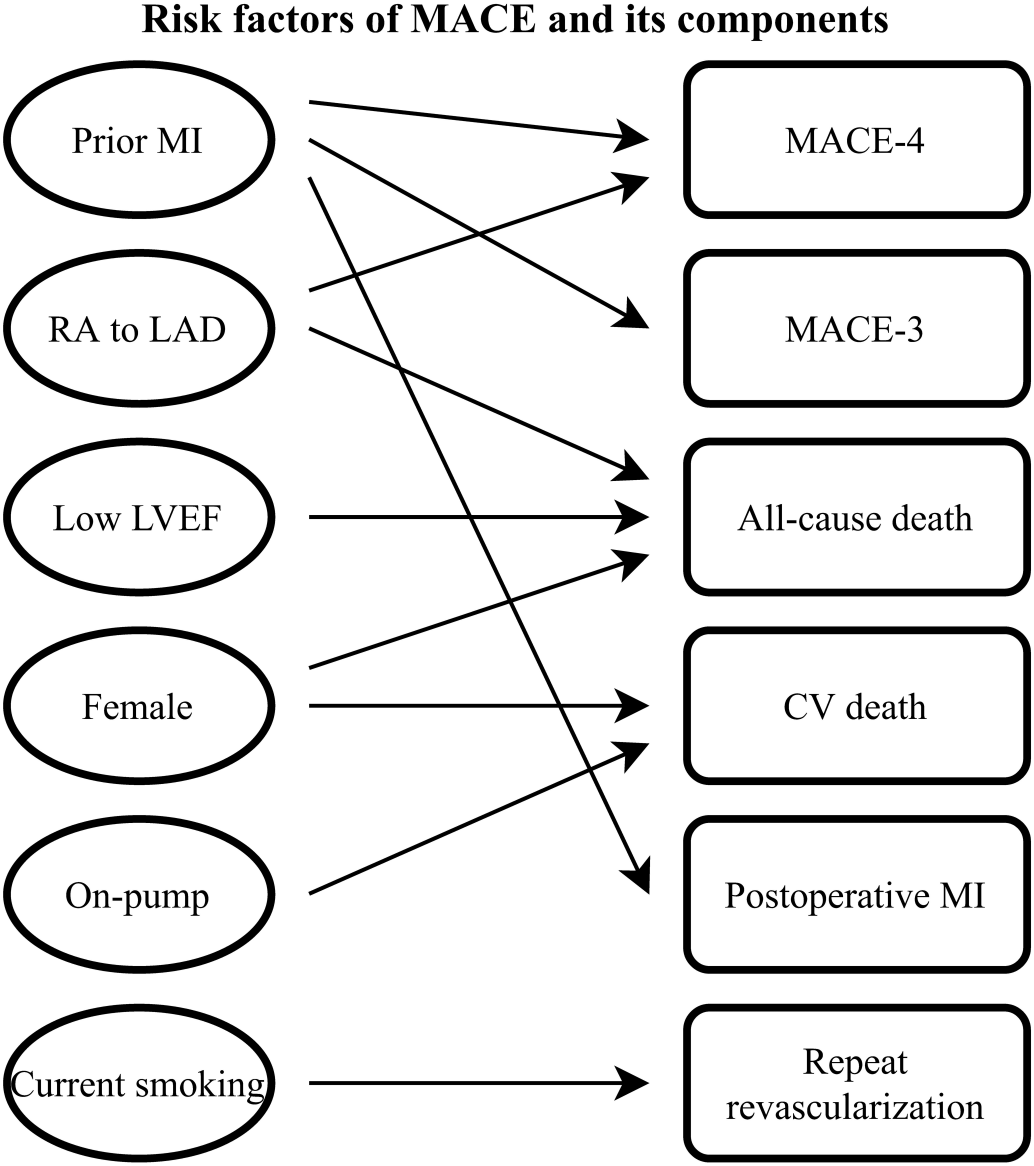
Risk factors of MACE and its components after RA-CABG. CV death, cardiovascular death; LAD, left anterior descending artery; LVEF, left ventricular ejection fraction; MACE, major adverse cardiovascular events; MI, myocardial infarction; RA, radial artery; RA-CABG, coronary artery bypass grafting using radial artery.

This case-control study focused on primary isolated RA-CABG patients during the last several years in our center and found prior MI together with RA to LAD as independent predictors for MACE-4; prior MI for MACE-3; female, LVEF < 40%, and RA to LAD for all-cause death; female and on-pump for CV-death; prior MI for postoperative MI and current smoking for repeat revascularization (Figure 2).

To compare the follow-up data with a meta-analysis which is composed of several randomized control trials, the definition of MACE-4 was adjusted accordingly (7). Then, a Kaplan-Meier curve was drawn, and the estimated incidence rates were comparable: 3.2% vs. 3.3%, 7.4% vs. 5.3%, 11.1% vs. 13.3% in 1 year, 3 years, and 5 years after RA-CABG (see Supplementary Figure S2).

Another case-control study that involved 1,613 patients is the Radial Artery 2,000, which merely explored risk factors of all-cause death after RA-CABG (14). In the perioperative period, both studies found being female, having MI at admission and low LVEF were risk factors of all-cause death. As for the early period, both studies revealed low LVEF and CKD as risk factors and CKD as an independent risk factor. Potential explanations for the discrepancies could be our stricter inclusion criteria and a relatively smaller sample size.

Our results were also compared with a retrospective cohort study with an averaged 8.1 years of follow-up duration focusing on predictors of mortality (15). In the current study, only LVEF < 40% was an independent risk factor, which corresponds with the lower LVEF suggested by the cohort study. Several independent risk factors revealed by the cohort study with the COX proportional hazards regression model, such as diabetes and hypertension, were not detected as independent risk factors by the present study. It is probably due to the lack of a longer observation, if not for the improved secondary prevention.

Meanwhile, low LVEF was also suggested as an independent predictor of 10-year mortality by another retrospective study (16). And in the univariate analysis part, being female was also found to be a potential risk factor of 10-year mortality.

The results of the present study might be a reference to a rational use of RA in CABG. For instance, prior MI could lead to MACE-4 or MI occurrence. Patients with prior MI could benefit more from CABG in lowering the MACE-4 incidence when RA rather than SVG was chosen as the second graft (7). Therefore, the higher MACE-4 incidence may not be associated with the application of RA but the MI history itself. In short, RA is still recommended in such patients.

LIMA has always been recommended and applied to bypass the LAD to improve survival and reduce recurrent ischemic events (17–22). For various reasons, some revascularizations of diseased LADs were not completed by LIMA. There are LADs of 50 patients who were not grafted by the LIMA. Among these, LIMAs of 35 patients were in poor condition. For the other 15 patients, the surgeries were conducted in the earlier years, and LIMAs were used to revascularize other severely diseased coronary arteries, for example, the diagonal arteries. Actually, the LIMA has been recommended to bypass the diseased LAD by the end of the last century. In alignment with this practice, our center also adopted the strategy of prioritizing LIMA grafting for diseased LAD before 2009 when the patients were included in the current study. And within the same timeframe, other patients did receive LIMA to LAD. So, the 15 patients were probably found to have a usable LIMA. But the operating surgeon determined that RA may better support the high flow of LAD because of the diameter limitation of LIMAs in these patients. Another consideration for not performing the exclusion is patients without a suitable LIMA to bypass LAD could arise from factors such as pre-existing LIMA disease due to diabetes or a limited LIMA diameter attributed to sex differences, especially in females. So, it should also be noted that the absence of a usable LIMA itself, rather than the use of RA as an alternative, could potentially serve as a risk factor for adverse events. Additionally, taking into account the restricted inclusion period and the single-center nature of the study, the act of exclusion could result in the loss of enrollment continuity and valuable data within the present study. Though the RA was used alternatively in the 50 patients, RA to LAD was still an indication for the occurrence of MACE-4, MACE-3, or all-cause death. In addition to LIMA, RA, RIMA, and SVG are used as grafts most often. When used to revascularize LAD, SVG was also inferior to LIMA (18). But when applied as the second graft, though RA and RIMA showed no difference in long-term clinical outcome, both outperformed the SVG (6). So, when LIMA is unavailable in CABG, the completion of revascularization of LAD through RIMA or RA warrants further investigation.

Low LVEF was an indication of all-cause death. But when compared to CABG with SVG as the second graft, RA-CABG tends to reduce the incidence of MACE-4 in either patients with LVEF ≥ 50% or patients with LVEF < 50% (7). Consequently, it may be reasonable to prioritize RA over SVG as the second graft.

Usually, males tend to have a survival advantage after receiving RA grafts because of a larger diameter resulting from sex differences (23). Being female was also indeed found to be an independent predictor for both all-cause death and CV-death in the current study. However, a different opinion proposed that females could benefit more from RA-CABG than using SVG as the second graft (7, 24). To sum up, even if females benefit less from RA-CABG than males, RA is still recommended for females due to its superiority over SVG.

Current smoking was independently associated with repeat revascularization. We proposed that current smoking patients had a higher probability of postoperative cigarettes consumption, which might incur repeat revascularization (25, 26). And grafts for CABG could suffer from peripheral vascular disease (PVD) caused by current smoking (27). After grafting, such previously existing inflammation may accelerate the pathological process in grafts, and thus a repeat revascularization will be needed.

Some candidate predictors in cardiac surgery risk models, however, were not identified as independent risk factors in the current study (28). For example, diabetes, anemia, and CKD were independent risk factors for CABG but not for RA-CABG as the current study suggested (12). This could be attributed to the risk dilution effect of the application of RA, as it was indicated that patients with diabetes or kidney insufficiency could benefit more from CABG using RA rather than SVG as the second graft (7, 29). Notably, a significantly stenosed left main coronary artery (LM) was a potential protective factor with OR < 1 for several clinical outcomes. This is because in the presence of stenosed LM, RAs are thought to be less affected by the competitive flow of the native coronary artery system, especially when most RAs are used to revascularize branches of LM. In summary, RA-CABG may be beneficial to patients with certain risk factors like diabetes, CKD, or stenosed LM.

If not contraindicated, CCB, diltiazem specifically, will be prescribed 3 days after the procedure, and most patients (approximately 90%) are asked to continue this for 6 months as common practice. Therefore, the conclusion suggested by an observational study, that CCB prescription is associated with a better clinical outcome, though very meaningful, was not proven by the current study (30).

Several limitations should be stated. First, this case-control study is subject to selection bias and other unmeasured confounding. Second, some medical records were missing in a small fraction of patients. For instance, prior MI is a risk factor for a few adverse events. However, when delving into specifics such as whether the area is affected by a prior MI was revascularized, even though it is very likely, precise data can't be provided. Third, the conclusions may be influenced by patient referral patterns and local medical management, for example, institutional off-pump preference, and therefore may not generalize to a larger population. Fourth, although with the accumulation of more than 10 years, the number of RA-CABG patients is still relatively limited. Hence, together with the absence of previous studies for reference, the limited data restrict the feasibility of matching.

## Data Availability

The raw data supporting the conclusions of this article will be made available by the authors, without undue reservation.
