# Supplementary material for: Risk factors for major adverse cardiovascular events after coronary artery bypass grafting using radial artery grafts
Source: Front Cardiovasc Med. 2023 Sep 27;10:1238161. doi: 10.3389/fcvm.2023.1238161 (PMC10565515; doi:10.3389/fcvm.2023.1238161)
Supplement: Supplementary file 1 [file Datasheet1.zip › Supplemetary data/Supplementary Figures S1 to S2.docx]

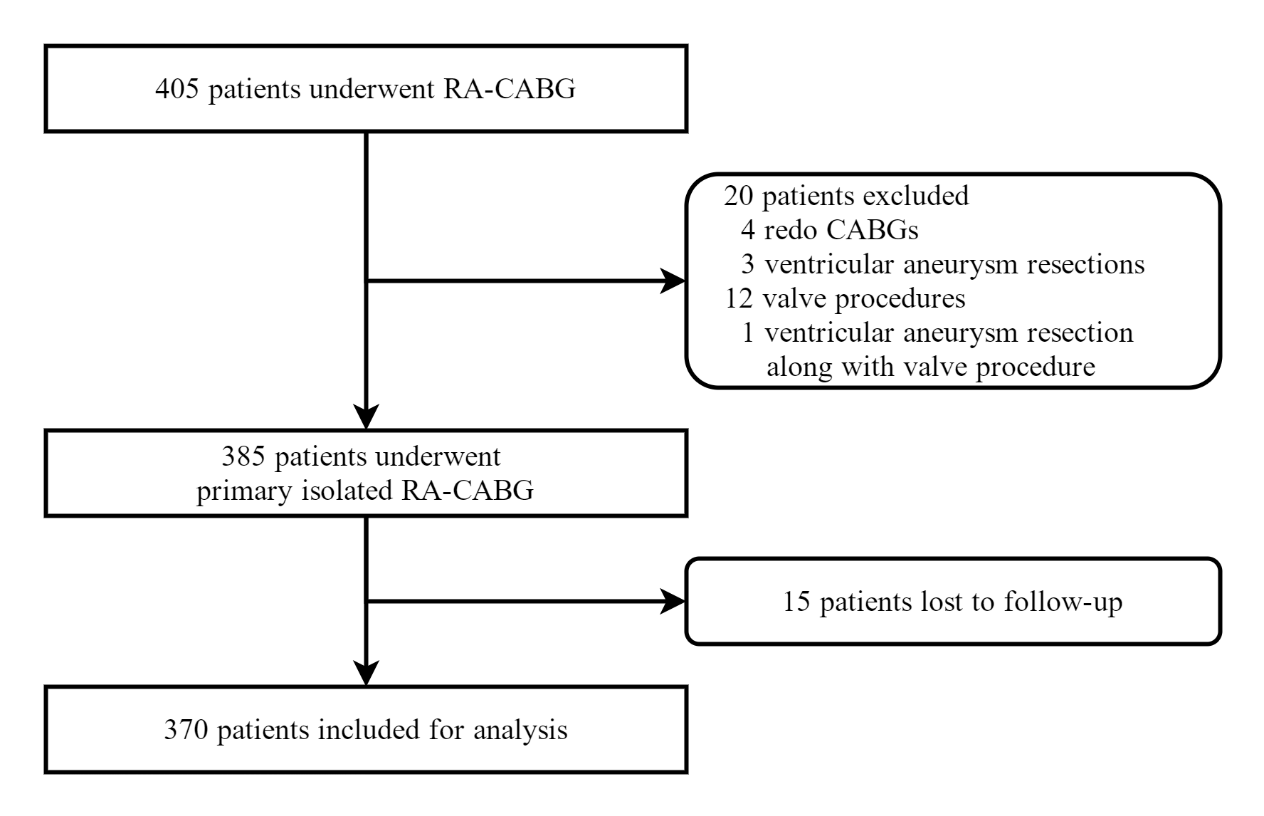


Figure S1. Flow of the Patients after the Application of Inclusion and Exclusion Criteria.

CABG, coronary artery bypass grafting; RA-CABG, coronary artery bypass grafting using radial artery.


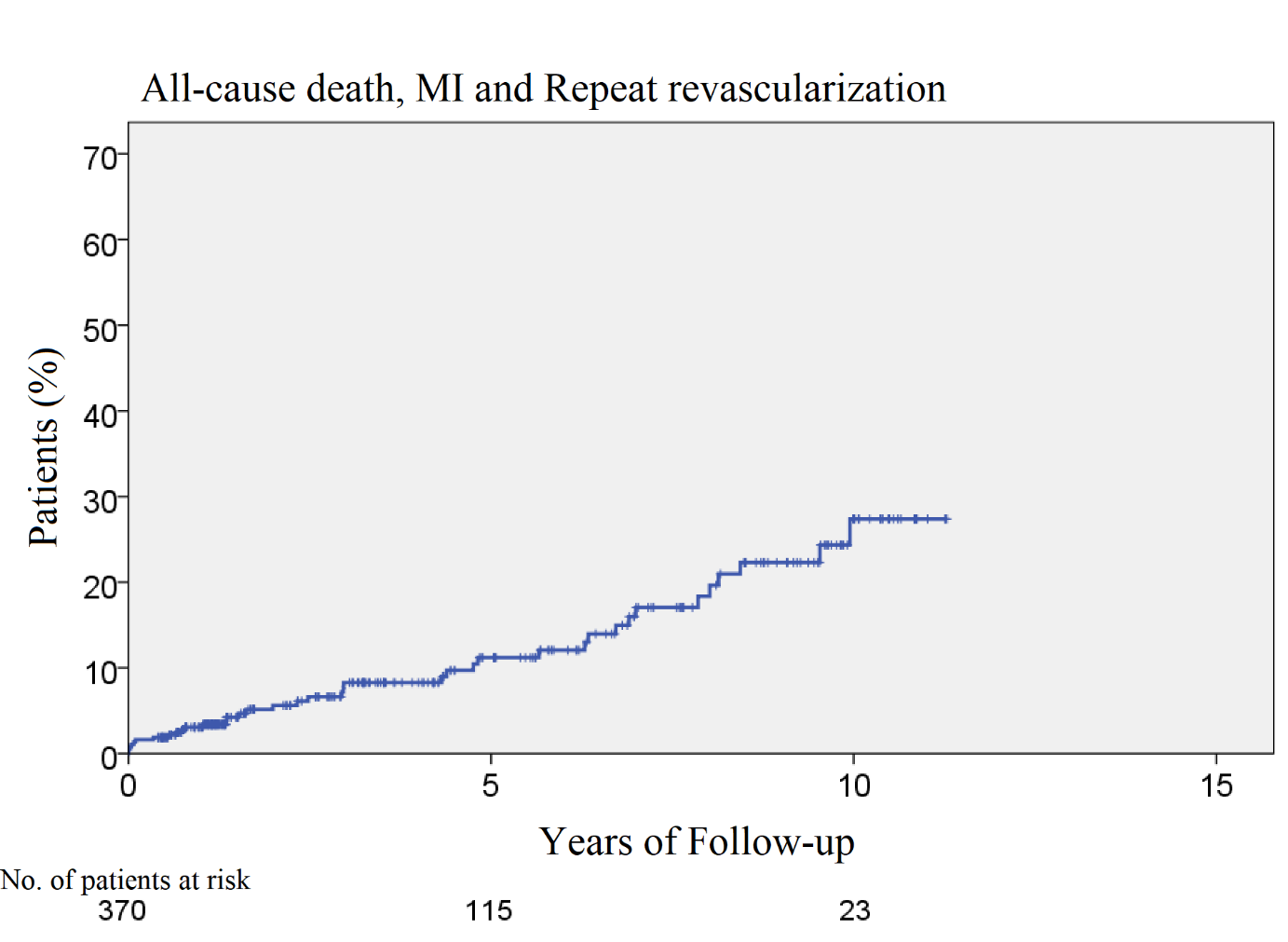


Figure S2. Cumulative incidence of the adjusted MACE which share the same definition with RADIAL.

MACE, major adverse cardiovascular events; MI, myocardial infarction.
