# Supplementary material for: Risk factors for major adverse cardiovascular events after coronary artery bypass grafting using radial artery grafts
Source: Front Cardiovasc Med. 2023 Sep 27;10:1238161. doi: 10.3389/fcvm.2023.1238161 (PMC10565515; doi:10.3389/fcvm.2023.1238161)
Supplement: Supplementary file 1 [file Datasheet1.zip › Supplemetary data/Supplementary Tables S1 to S22.docx]

Abbreviations and acronyms in the tables.

ACS, acute coronary syndrome; BIMA, bilateral internal mammary arteries; BMI, body mass index; CCB, calcium channel blockers; CKD, chronic kidney disease; COPD, chronic obstructive pulmonary disease; CV-death, cardiovascular death; LAD, left anterior descending artery; LIMA, left internal mammary artery; LM, left main coronary artery; LVEDD, left ventricular end diastolic diameter; LVEF, left ventricular ejection fraction; LVESD, left ventricular end systolic diameter; MI, myocardial infarction; NYHA, New York Heart Association; Pro-BNP, pro-B-type natriuretic peptide; PVD, peripheral vascular disease; RA, radial artery; TAR, total arterial revascularization.

Some definitions in the current study.

Abnormal platelet count: patients will be labelled with abnormal platelet count if platelet count <100*10^9 or >300*10^9 was reported in the most recent preoperative blood test.

Anemia: patients will be labelled with anemia if it was reported in preoperative medical records. Alternatively, hemoglobin<130g/L for male or hemoglobin<120g/L for female was reported in the most recent preoperative blood test.

Arterial grafts<50%: patients will be labelled with arterial grafts<50% if less than half of anastomoses in diseased coronary arteries were completed by arterial grafts.

Calcium channel blockers (CCB) for 6 months: patients will be labelled with CCB for 6 months if any kind of CCB was prescribed and was asked to continue for 6 months or more.

Chronic kidney disease (CKD): Patients will be labelled with CKD if it was reported in perioperative medical records, or glomerular filtration rate<60ml/min*1.73m^2 was reported in preoperative blood test.

Current smoking: Patients will be labelled with current smoking if patients didn’t quit smoking or had not quitted for more than a year at admission.

Diabetes mellitus: Patients will be labelled with diabetes mellitus if it was reported in perioperative medical records, or HbA1c≥6.5% was reported in preoperative blood test.

Hyperlipemia: Patients will be labelled with hyperlipemia if it was reported in perioperative medical records, or low-density lipoprotein≥2.6mmol/L was reported in preoperative blood test.

Incomplete revascularization: patients will be labelled with incomplete revascularization if any one of three coronary artery systems (left anterior descending artery system, left circumflex artery system and right coronary system) was not revascularized by any graft in the presence of significant stenosis (≥50%) in it.

Left main coronary artery (LM) stenosis: patients will be labelled with LM stenosis if a stenosis of 50% or more in LM was recorded in preoperative coronary angiography.

Non-bilateral internal mammary arteries (BIMA): patients will be labelled with non-BIMA if not both BIMA are used for revascularization.

Non-elective operation: patients will be labelled with non-elective operation if coronary artery bypass grafting was carried out in less than 3 days or more than 7 days since admission.

Non-total arterial revascularization (TAR): patients will be labelled with non-TAR if not all grafts for revascularization are arterial conduits.

Peripheral vascular disease (PVD): Patients will be labelled with PVD if it was reported in perioperative medical records, or any stenosis of 50% or more was reported in carotid arteries, vertebral arteries or femoral arteries by preoperative ultrasonography or angiography examination.

Prior myocardial infarction (MI): Patients will be labelled with prior MI if a history of MI was reported in perioperative medical records, or reduced ventricular wall motion was reported in preoperative ultrasound cardiography.

Radial artery grafted to diseased left anterior descending artery (RA to LAD): patients will be labelled with RA to LAD (non-LIMA to LAD) if RA rather than LIMA was used to revascularize diseased LAD.

Three-system disease: patients will be labelled with three-system disease if all three coronary artery systems (left anterior descending artery system, left circumflex artery system and right coronary system) have significant stenosed (≥50%) coronary arteries in it.

Table S1-S22 are statistical analysis for various outcomes.

Table S1. Univariate and Multivariate Logistic Regression Analysis of Risk Factors for MACE-4

|  | Univariate Estimates | | |  | Multivariate Estimates | | |
| --- | --- | --- | --- | --- | --- | --- | --- |
|  | OR | 95% CI | P |  | OR | 95% CI | P |
| **Preoperative factors** |  |  |  |  |  |  |  |
| Female | 1.11 | 0.51-2.40 | .80 |  |  |  |  |
| BMI ≥24 | 0.87 | 0.54-1.39 | .55 |  |  |  |  |
| BMI ≥28 | 1.40 | 0.79-2.48 | .25 |  |  |  |  |
| Aged 60 years or more | 1.20 | 0.75-1.94 | .45 |  |  |  |  |
| Aged 65 years or more | 1.30 | 0.72-2.38 | .39 |  |  |  |  |
| Aged 70 years or more | 2.62 | 1.15-5.94 | .02 |  | 2.14 | 0.69-6.63 | .19 |
| ACS at admission | 1.03 | 0.63-1.69 | .90 |  |  |  |  |
| MI at admission | 2.35 | 1.19-4.67 | .01 |  | 1.50 | 0.61-3.67 | .38 |
| Diabetes mellitus | 0.96 | 0.60-1.54 | .85 |  |  |  |  |
| Hypertension | 1.24 | 0.72-2.13 | .45 |  |  |  |  |
| COPD | 2.12 | 0.55-8.13 | .27 |  |  |  |  |
| Hyperlipemia | 0.73 | 0.46-1.15 | .18 |  |  |  |  |
| CKD | 1.51 | 0.67-3.39 | .32 |  |  |  |  |
| PVD | 1.00 | 0.40-2.48 | >.99 |  |  |  |  |
| Prior MI | 2.69 | 1.68-4.30 | <.001 |  | 2.20 | 1.09-4.43 | .03 |
| Prior stroke | 1.13 | 0.53-2.37 | .76 |  |  |  |  |
| Smoking history | 1.26 | 0.78-2.05 | .34 |  |  |  |  |
| Current smoking | 1.59 | 1.01-2.53 | .05 |  | 1.39 | 0.75-2.60 | .30 |
| NYHA classification III or IV | 0.87 | 0.54-1.41 | .56 |  |  |  |  |
| Pro-BNP>600pg/ml | 2.28 | 1.13-4.61 | .02 |  | 1.25 | 0.49-3.17 | .65 |
| LVESD>40mm | 3.92 | 1.87-8.22 | <.001 |  | 2.78 | 0.63-12.26 | .18 |
| LVEDD>60mm | 3.20 | 1.13-9.05 | .03 |  | 0.94 | 0.17-5.31 | .95 |
| LVEF<40% | 2.72 | 0.67-11.09 | .16 |  |  |  |  |
| LVEF<50% | 2.34 | 1.16-4.71 | .02 |  | 0.59 | 0.13-2.63 | .49 |
| Anemia | 1.34 | 0.80-2.26 | .27 |  |  |  |  |
| Abnormal platelet count | 1.23 | 0.58-2.61 | .59 |  |  |  |  |
| LM stenosis | 0.76 | 0.45-1.28 | .30 |  |  |  |  |
| Three-system disease | 1.30 | 0.77-2.18 | .32 |  |  |  |  |
| **Surgical factors** |  |  |  |  |  |  |  |
| Non-elective operation | 1.17 | 0.71-1.92 | .55 |  |  |  |  |
| RA to LAD (non-LIMA to LAD) | 3.75 | 1.53-9.22 | .004 |  | 4.33 | 1.18-15.89 | .03 |
| Non-BIMA | 2.33 | 0.52-10.49 | .27 |  |  |  |  |
| Stenosis of RA targeted coronary artery<70% | 2.13 | 0.77-5.88 | .14 |  |  |  |  |
| Arterial grafts<50% | 2.70 | 0.66-11.03 | .17 |  |  |  |  |
| Non-TAR | 1.33 | 0.83-2.13 | .23 |  |  |  |  |
| Incomplete revascularization | 1.41 | 0.83-2.13 | .20 |  |  |  |  |
| On-pump | 8.31 | 1.65-41.88 | .01 |  | - | - | .98 |
| **Postoperative Medication** |  |  |  |  |  |  |  |
| CCB for 6 months | 1.06 | 0.53-2.15 | .86 |  |  |  |  |

Table S2. Univariate and Multivariate Logistic Regression Analysis of Risk Factors for MACE-3

|  | Univariate Estimates | | |  | Multivariate Estimates | | |
| --- | --- | --- | --- | --- | --- | --- | --- |
|  | OR | 95% CI | P |  | OR | 95% CI | P |
| **Preoperative factors** |  |  |  |  |  |  |  |
| Female | 1.07 | 0.47-2.46 | .87 |  |  |  |  |
| BMI ≥24 | 0.93 | 0.56-1.55 | .78 |  |  |  |  |
| BMI ≥28 | 1.52 | 0.83-2.76 | .17 |  |  |  |  |
| Aged 60 years or more | 1.10 | 0.66-1.84 | .71 |  |  |  |  |
| Aged 65 years or more | 1.22 | 0.64-2.32 | .55 |  |  |  |  |
| Aged 70 years or more | 2.48 | 1.07-5.76 | .03 |  | 2.71 | 0.81-9.06 | .11 |
| ACS at admission | 1.11 | 0.65-1.89 | .69 |  |  |  |  |
| MI at admission | 2.88 | 1.43-5.78 | .003 |  | 1.49 | 0.59-3.79 | .40 |
| Diabetes mellitus | 1.02 | 0.61-1.69 | .95 |  |  |  |  |
| Hypertension | 1.11 | 0.62-1.97 | .73 |  |  |  |  |
| COPD | 2.89 | 0.75-11.12 | .12 |  |  |  |  |
| Hyperlipemia | 0.77 | 0.47-1.25 | .29 |  |  |  |  |
| CKD | 1.17 | 0.48-2.85 | .74 |  |  |  |  |
| PVD | 1.25 | 0.50-3.12 | .63 |  |  |  |  |
| Prior MI | 3.48 | 2.10-5.78 | <.001 |  | 3.10 | 1.41-6.83 | .005 |
| Prior stroke | 1.13 | 0.51-2.49 | .77 |  |  |  |  |
| Smoking history | 1.07 | 0.64-1.79 | .78 |  |  |  |  |
| Current smoking | 1.45 | 0.89-2.37 | .14 |  |  |  |  |
| NYHA classification III or IV | 1.08 | 0.64-1.82 | .79 |  |  |  |  |
| Pro-BNP>600pg/ml | 2.87 | 1.41-5.85 | .004 |  | 1.32 | 0.50-3.52 | .58 |
| LVESD>40mm | 4.64 | 2.20-9.76 | <.001 |  | 3.19 | 0.70-14.58 | .13 |
| LVEDD>60mm | 3.19 | 1.12-9.07 | .03 |  | 0.64 | 0.11-3.81 | .62 |
| LVEF<40% | 3.57 | 0.87-14.59 | .08 |  | 0.48 | 0.05-4.91 | .54 |
| LVEF<50% | 2.78 | 1.36-5.69 | .005 |  | 0.76 | 0.17-3.39 | .72 |
| Anemia | 1.23 | 0.70-2.16 | .47 |  |  |  |  |
| Abnormal platelet count | 1.22 | 0.55-2.72 | .63 |  |  |  |  |
| LM stenosis | 0.53 | 0.29-0.97 | .04 |  | 0.51 | 0.23-1.13 | .10 |
| Three-system disease | 1.52 | 0.86-2.68 | .15 |  |  |  |  |
| **Surgical factors** |  |  |  |  |  |  |  |
| Non-elective operation | 1.31 | 0.78-2.23 | .31 |  |  |  |  |
| RA to LAD (non-LIMA to LAD) | 2.76 | 1.12-6.82 | .03 |  | 1.81 | 0.45-7.30 | .41 |
| Non-BIMA | 1.75 | 0.39-8.33 | .46 |  |  |  |  |
| Stenosis of RA targeted coronary artery<70% | 2.17 | 0.76-6.24 | .15 |  |  |  |  |
| Arterial grafts<50% | 3.57 | 0.88-14.54 | .08 |  | 1.01 | 0.10-9.77 | >.99 |
| Non-TAR | 1.82 | 1.12-2.94 | .02 |  | 1.06 | 0.54-2.08 | .94 |
| Incomplete revascularization | 1.15 | 0.64-2.04 | .65 |  |  |  |  |
| On-pump | 6.07 | 1.42-25.94 | .02 |  | 8.91 | 0.52-153.68 | .13 |
| **Postoperative Medication** |  |  |  |  |  |  |  |
| CCB for 6 months | 1.03 | 0.49-2.17 | .95 |  |  |  |  |

Table S3. Univariate and Multivariate Logistic Regression Analysis of Risk Factors for All-cause death

|  | Univariate Estimates | | |  | Multivariate Estimates | | |
| --- | --- | --- | --- | --- | --- | --- | --- |
|  | OR | 95% CI | P |  | OR | 95% CI | P |
| **Preoperative factors** |  |  |  |  |  |  |  |
| Female | 3.21 | 1.20-8.62 | .02 |  | 6.12 | 1.13-33.32 | .04 |
| BMI ≥24 | 1.08 | 0.47-2.47 | .86 |  |  |  |  |
| BMI ≥28 | 1.35 | 0.52-3.48 | .54 |  |  |  |  |
| Aged 60 years or more | 2.72 | 1.23-6.01 | .01 |  | 4.49 | 0.62-32.27 | .14 |
| Aged 65 years or more | 2.93 | 1.25-6.89 | .01 |  | 0.90 | 0.09-8.53 | .92 |
| Aged 70 years or more | 6.32 | 2.36-16.89 | <.001 |  | 0.59 | 0.03-10.07 | .71 |
| ACS at admission | 1.37 | 0.56-3.33 | .49 |  |  |  |  |
| MI at admission | 1.10 | 0.32-3.84 | .88 |  |  |  |  |
| Diabetes mellitus | 1.40 | 0.63-3.08 | .41 |  |  |  |  |
| Hypertension | 1.45 | 0.53-3.95 | .47 |  |  |  |  |
| COPD | 1.81 | 0.21-15.32 | .59 |  |  |  |  |
| Hyperlipemia | 0.84 | 0.38-1.86 | .66 |  |  |  |  |
| CKD | 4.17 | 1.53-11.39 | .005 |  | 1.85 | 0.25-13.46 | .55 |
| PVD | 1.19 | 0.26-5.41 | .83 |  |  |  |  |
| Prior MI | 1.73 | 0.79-3.79 | .17 |  |  |  |  |
| Prior stroke | 1.14 | 0.33-3.97 | .84 |  |  |  |  |
| Smoking history | 0.81 | 0.37-1.81 | .61 |  |  |  |  |
| Current smoking | 0.94 | 0.43-2.07 | .89 |  |  |  |  |
| NYHA classification III or IV | 0.36 | 0.16-0.82 | .02 |  | 1.92 | 0.31-11.87 | .48 |
| Pro-BNP>600pg/ml | 3.65 | 1.16-11.53 | .03 |  | 3.74 | 0.56-24.78 | .17 |
| LVESD>40mm | 3.65 | 1.35-9.87 | .01 |  | - | - | .84 |
| LVEDD>60mm | 3.59 | 0.94-13.62 | .06 |  | - | - | .81 |
| LVEF<40% | 15.41 | 3.61-65.79 | <.001 |  | 86.22 | - | .07 |
| LVEF<50% | 3.13 | 1.17-8.39 | .02 |  | 0.28 | 0.01-15.03 | .53 |
| Anemia | 2.03 | 0.89-4.62 | .09 |  | 0.62 | 0.10-3.87 | .61 |
| Abnormal platelet count | 1.21 | 0.35-4.26 | .76 |  |  |  |  |
| LM stenosis | 1.20 | 0.51-2.80 | .68 |  |  |  |  |
| Three-system disease | 0.87 | 0.37-2.04 | .75 |  |  |  |  |
| **Surgical factors** |  |  |  |  |  |  |  |
| Non-elective operation | 1.56 | 0.69-3.52 | .29 |  |  |  |  |
| RA to LAD (non-LIMA to LAD) | 3.44 | 1.06-11.14 | .04 |  | 5.42 | 0.50-58.45 | .16 |
| Non-BIMA | - | - | .98 |  |  |  |  |
| Stenosis of RA targeted coronary artery<70% | 1.85 | 0.40-8.33 | .43 |  |  |  |  |
| Arterial grafts<50% | 4.55 | 0.86-23.97 | .07 |  | - | - | .93 |
| Non-TAR | 1.27 | 0.57-2.78 | .56 |  |  |  |  |
| Incomplete revascularization | 0.82 | 0.30-2.27 | .70 |  |  |  |  |
| On-pump | 25.76 | 5.78-114.85 | <.001 |  | - | - | >.99 |
| **Postoperative Medication** |  |  |  |  |  |  |  |
| CCB for 6 months | 1.51 | 0.34-6.68 | .83 |  | - | - | >.99 |

Table S4. Univariate and Adjusted Multivariate Logistic Regression Analysis of Risk Factors for All-cause Death ^a^

|  | Univariate Estimates | | |  | Adjusted Estimates | | |
| --- | --- | --- | --- | --- | --- | --- | --- |
|  | OR | 95% CI | P |  | OR | 95% CI | P |
| **Preoperative factors** |  |  |  |  |  |  |  |
| Female | 3.21 | 1.20-8.62 | .02 |  | 4.53 | 1.06-19.41 | .04 |
| BMI ≥24 | 1.08 | 0.47-2.47 | .86 |  |  |  |  |
| BMI ≥28 | 1.35 | 0.52-3.48 | .54 |  |  |  |  |
| Aged 60 years or more | 2.72 | 1.23-6.01 | .01 |  | 1.78 | 0.34-9.32 | .49 |
| Aged 65 years or more | 2.93 | 1.25-6.89 | .01 |  | 0.83 | 0.10-7.18 | .87 |
| Aged 70 years or more | 6.32 | 2.36-16.89 | <.001 |  | 1.24 | 0.11-13.98 | .86 |
| ACS at admission | 1.37 | 0.56-3.33 | .49 |  |  |  |  |
| MI at admission | 1.10 | 0.32-3.84 | .88 |  |  |  |  |
| Diabetes mellitus | 1.40 | 0.63-3.08 | .41 |  |  |  |  |
| Hypertension | 1.45 | 0.53-3.95 | .47 |  |  |  |  |
| COPD | 1.81 | 0.21-15.32 | .59 |  |  |  |  |
| Hyperlipemia | 0.84 | 0.38-1.86 | .66 |  |  |  |  |
| CKD | 4.17 | 1.53-11.39 | .005 |  | 2.32 | 0.40-13.25 | .35 |
| PVD | 1.19 | 0.26-5.41 | .83 |  |  |  |  |
| Prior MI | 1.73 | 0.79-3.79 | .17 |  |  |  |  |
| Prior stroke | 1.14 | 0.33-3.97 | .84 |  |  |  |  |
| Smoking history | 0.81 | 0.37-1.81 | .61 |  |  |  |  |
| Current smoking | 0.94 | 0.43-2.07 | .89 |  |  |  |  |
| NYHA classification III or IV | 0.36 | 0.16-0.82 | .02 |  | 0.93 | 0.25-3.45 | .92 |
| Pro-BNP>600pg/ml | 3.65 | 1.16-11.53 | .03 |  | 2.19 | 0.47-10.11 | .32 |
| LVESD>40mm | 3.65 | 1.35-9.87 | .01 |  |  |  |  |
| LVEDD>60mm | 3.59 | 0.94-13.62 | .06 |  |  |  |  |
| LVEF<40% | 15.41 | 3.61-65.79 | <.001 |  | 21.00 | 1.20-368.35 | .04 |
| LVEF<50% | 3.13 | 1.17-8.39 | .02 |  | 1.25 | 0.14-11.07 | .84 |
| Anemia | 2.03 | 0.89-4.62 | .09 |  | 0.69 | 0.15-3.24 | .64 |
| Abnormal platelet count | 1.21 | 0.35-4.26 | .76 |  |  |  |  |
| LM stenosis | 1.20 | 0.51-2.80 | .68 |  |  |  |  |
| Three-system disease | 0.87 | 0.37-2.04 | .75 |  |  |  |  |
| **Surgical factors** |  |  |  |  |  |  |  |
| Non-elective operation | 1.56 | 0.69-3.52 | .29 |  |  |  |  |
| RA to LAD (non-LIMA to LAD) | 3.44 | 1.06-11.14 | .04 |  | 8.55 | 1.35-54.10 | .02 |
| Non-BIMA | - | - | .98 |  |  |  |  |
| Stenosis of RA targeted coronary artery<70% | 1.85 | 0.40-8.33 | .43 |  |  |  |  |
| Arterial grafts<50% | 4.55 | 0.86-23.97 | .07 |  |  |  |  |
| Non-TAR | 1.27 | 0.57-2.78 | .56 |  |  |  |  |
| Incomplete revascularization | 0.82 | 0.30-2.27 | .70 |  |  |  |  |
| On-pump | 25.76 | 5.78-114.85 | <.001 |  |  |  |  |
| **Postoperative Medication** |  |  |  |  |  |  |  |
| CCB for 6 months | 1.51 | 0.34-6.68 | .83 |  |  |  |  |

^a^ Multivariable analysis was conducted again after excluding the variables without astringency from the Prior multivariate logistic regression modeling

Table S5. Univariate and Multivariate Logistic Regression Analysis of Risk Factors for CV-death

|  | Univariate Estimates | | |  | Multivariate Estimates | | |
| --- | --- | --- | --- | --- | --- | --- | --- |
|  | OR | 95% CI | P |  | OR | 95% CI | P |
| **Preoperative factors** |  |  |  |  |  |  |  |
| Female | 3.07 | 0.95-9.91 | .06 |  | 8.28 | 1.06-64.62 | .04 |
| BMI ≥24 | 1.07 | 0.39-2.93 | .89 |  |  |  |  |
| BMI ≥28 | 1.83 | 0.63-5.34 | .27 |  |  |  |  |
| Aged 60 years or more | 2.09 | 0.81-5.40 | .13 |  |  |  |  |
| Aged 65 years or more | 2.82 | 1.01-7.84 | .05 |  | 1.04 | 0.08-13.78 | .98 |
| Aged 70 years or more | 6.38 | 2.07-19.68 | .001 |  | 2.35 | 0.10-56.50 | .60 |
| ACS at admission | 1.68 | 0.54-5.21 | .37 |  |  |  |  |
| MI at admission | 1.81 | 0.50-6.57 | .37 |  |  |  |  |
| Diabetes mellitus | 1.09 | 0.41-2.87 | .87 |  |  |  |  |
| Hypertension | 1.13 | 0.36-3.53 | .83 |  |  |  |  |
| COPD | 2.86 | 0.33-24.95 | .34 |  |  |  |  |
| Hyperlipemia | 0.68 | 0.25-1.83 | .45 |  |  |  |  |
| CKD | 3.90 | 1.19-12.78 | .02 |  | 4.30 | 0.46-39.96 | .20 |
| PVD | 1.77 | 0.38-8.34 | .47 |  |  |  |  |
| Prior MI | 2.96 | 1.12-7.84 | .03 |  | 2.64 | 0.30-23.25 | .38 |
| Prior stroke | 0.52 | 0.07-4.00 | .53 |  |  |  |  |
| Smoking history | 0.70 | 0.27-1.81 | .46 |  |  |  |  |
| Current smoking | 1.29 | 0.50-3.35 | .60 |  |  |  |  |
| NYHA classification III or IV | 0.46 | 0.17-1.22 | .12 |  |  |  |  |
| Pro-BNP>600pg/ml | 5.55 | 1.61-19.15 | .007 |  | 2.77 | 0.38-20.24 | .32 |
| LVESD>40mm | 6.23 | 2.16-17.95 | .001 |  | 0.32 | 0.00-22.03 | .60 |
| LVEDD>60mm | 5.63 | 1.44-22.10 | .01 |  | 4.05 | 0.12-136.02 | .44 |
| LVEF<40% | 24.78 | 5.61-109.45 | <.001 |  | 8.95 | 0.28-286.37 | .22 |
| LVEF<50% | 5.35 | 1.87-15.27 | .002 |  | 1.81 | 0.07-44.95 | .72 |
| Anemia | 1.67 | 0.61-4.60 | .32 |  |  |  |  |
| Abnormal platelet count | 1.21 | 0.27-5.49 | .81 |  |  |  |  |
| LM stenosis | 1.15 | 0.42-3.21 | .78 |  |  |  |  |
| Three-system disease | 1.20 | 0.41-3.49 | .74 |  |  |  |  |
| **Surgical factors** |  |  |  |  |  |  |  |
| Non-elective operation | 1.67 | 0.63-4.44 | .30 |  |  |  |  |
| RA to LAD (non-LIMA to LAD) | 4.04 | 1.06-15.46 | .04 |  | 2.88 | 0.18-46.91 | .46 |
| Non-BIMA | - | - | .98 |  |  |  |  |
| Stenosis of RA targeted coronary artery<70% | 1.28 | 0.16-10.37 | .82 |  |  |  |  |
| Arterial grafts<50% | 7.14 | 1.35-37.76 | .02 |  | 2.86 | 0.04-223.18 | .64 |
| Non-TAR | 2.56 | 0.97-6.67 | .06 |  | 0.47 | 0.08-2.78 | .40 |
| Incomplete revascularization | 0.42 | 0.10-1.89 | .26 |  |  |  |  |
| On-pump | 24.85 | 5.63-109.76 | <.001 |  | 105.6 | 3.66-3044.7 | .007 |
| **Postoperative Medication** |  |  |  |  |  |  |  |
| CCB for 6 months | 1.96 | 0.25-15.34 | .81 |  |  |  |  |

Table S6. Univariate and Multivariate Logistic Regression Analysis of Risk Factors for MI

|  | Univariate Estimates | | |  | Adjusted Estimates | | |
| --- | --- | --- | --- | --- | --- | --- | --- |
|  | OR | 95% CI | P |  | OR | 95% CI | P |
| **Preoperative factors** |  |  |  |  |  |  |  |
| Female | 0.78 | 0.29-2.09 | .62 |  |  |  |  |
| BMI ≥24 | 0.85 | 0.49-1.48 | .57 |  |  |  |  |
| BMI ≥28 | 1.63 | 0.86-3.08 | .14 |  |  |  |  |
| Aged 60 years or more | 1.00 | 0.57-1.77 | .99 |  |  |  |  |
| Aged 65 years or more | 1.07 | 0.52-2.18 | .86 |  |  |  |  |
| Aged 70 years or more | 1.89 | 0.75-4.72 | .18 |  |  |  |  |
| ACS at admission | 1.09 | 0.61-1.95 | .76 |  |  |  |  |
| MI at admission | 3.59 | 1.76-7.35 | <.001 |  | 2.54 | 1.00-6.45 | .05 |
| Diabetes mellitus | 1.05 | 0.60-1.81 | .87 |  |  |  |  |
| Hypertension | 0.91 | 0.49-1.68 | .77 |  |  |  |  |
| COPD | 2.36 | 0.57-9.78 | .24 |  |  |  |  |
| Hyperlipemia | 0.82 | 0.48-1.40 | .46 |  |  |  |  |
| CKD | 0.75 | 0.25-2.25 | .61 |  |  |  |  |
| PVD | 1.61 | 0.64-4.06 | .31 |  |  |  |  |
| Prior MI | 3.78 | 2.17-6.58 | <.001 |  | 3.11 | 1.40-6.94 | .006 |
| Prior stroke | 1.31 | 0.57-3.01 | .53 |  |  |  |  |
| Smoking history | 1.17 | 0.66-2.05 | .59 |  |  |  |  |
| Current smoking | 1.38 | 0.81-2.36 | .24 |  |  |  |  |
| NYHA classification III or IV | 0.98 | 0.56-1.72 | .94 |  |  |  |  |
| Pro-BNP>600pg/ml | 2.55 | 1.21-5.38 | .01 |  | 1.08 | 0.39-2.96 | .89 |
| LVESD>40mm | 4.25 | 1.99-9.07 | <.001 |  | 3.42 | 0.86-13.57 | .08 |
| LVEDD>60mm | 2.39 | 0.79-7.25 | .12 |  |  |  |  |
| LVEF<40% | 1.55 | 0.31-7.84 | .60 |  |  |  |  |
| LVEF<50% | 2.58 | 1.22-5.48 | .01 |  | 0.54 | 0.13-2.21 | .39 |
| Anemia | 1.16 | 0.63-2.14 | .64 |  |  |  |  |
| Abnormal platelet count | 1.42 | 0.61-3.27 | .42 |  |  |  |  |
| LM stenosis | 0.41 | 0.21-0.83 | .01 |  | 0.46 | 0.19-1.10 | .08 |
| Three-system disease | 2.00 | 1.03-3.87 | .04 |  | 1.76 | 0.67-4.61 | .25 |
| **Surgical factors** |  |  |  |  |  |  |  |
| Non-elective operation | 1.35 | 0.76-2.40 | .30 |  |  |  |  |
| RA to LAD (non-LIMA to LAD) | 2.38 | 0.92-6.15 | .07 |  | 1.60 | 0.40-6.39 | .50 |
| Non-BIMA | 1.32 | 0.29-5.88 | .72 |  |  |  |  |
| Stenosis of RA targeted coronary artery<70% | 2.22 | 0.74-6.72 | .16 |  |  |  |  |
| Arterial grafts<50% | 2.86 | 0.66-12.32 | .16 |  |  |  |  |
| Non-TAR | 2.00 | 1.16-3.45 | .01 |  | 1.09 | 0.52-2.27 | .82 |
| Incomplete revascularization | 1.54 | 0.84-2.78 | .16 |  |  |  |  |
| On-pump | 2.85 | 0.66-12.22 | .16 |  |  |  |  |
| **Postoperative Medication** |  |  |  |  |  |  |  |
| CCB for 6 months | 1.02 | 0.45-2.30 | .96 |  |  |  |  |

Table S7. Univariate and Multivariate Logistic Regression Analysis of Risk Factors for Stroke

|  | Univariate Estimates | | |  | Adjusted Estimates | | |
| --- | --- | --- | --- | --- | --- | --- | --- |
|  | OR | 95% CI | P |  | OR | 95% CI | P |
| **Preoperative factors** |  |  |  |  |  |  |  |
| Female | 3.52 | 0.90-13.67 | .07 |  | - | - | - |
| BMI ≥24 | 1.63 | 0.43-6.13 | .47 |  |  |  |  |
| BMI ≥28 | 1.56 | 0.41-5.93 | .51 |  |  |  |  |
| Aged 60 years or more | 2.06 | 0.65-6.52 | .22 |  |  |  |  |
| Aged 65 years or more | 1.80 | 0.47-6.85 | .39 |  |  |  |  |
| Aged 70 years or more | 2.91 | 0.60-14.08 | .18 |  |  |  |  |
| ACS at admission | 0.65 | 0.20-2.08 | .46 |  |  |  |  |
| MI at admission | - | - | .97 |  |  |  |  |
| Diabetes mellitus | 1.22 | 0.38-3.93 | .74 |  |  |  |  |
| Hypertension | 3.64 | 0.46-28.59 | .22 |  |  |  |  |
| COPD | 3.98 | 0.44-35.77 | .22 |  |  |  |  |
| Hyperlipemia | 0.99 | 0.31-3.12 | .99 |  |  |  |  |
| CKD | 2.55 | 0.53-12.27 | .24 |  |  |  |  |
| PVD | - | - | .97 |  |  |  |  |
| Prior MI | 1.28 | 0.40-4.13 | .68 |  |  |  |  |
| Prior stroke | - | - | .97 |  |  |  |  |
| Smoking history | 0.39 | 0.12-1.26 | .12 |  |  |  |  |
| Current smoking | 0.72 | 0.23-2.32 | .59 |  |  |  |  |
| NYHA classification III or IV | 5.56 | 0.70-43.83 | .10 |  |  |  |  |
| Pro-BNP>600pg/ml | 3.09 | 0.55-17.47 | .20 |  |  |  |  |
| LVESD>40mm | - | - | .98 |  |  |  |  |
| LVEDD>60mm | - | - | .98 |  |  |  |  |
| LVEF<40% | - | - | .99 |  |  |  |  |
| LVEF<50% | - | - | .97 |  |  |  |  |
| Anemia | 1.66 | 0.49-5.64 | .42 |  |  |  |  |
| Abnormal platelet count | 0.87 | 0.11-6.92 | .89 |  |  |  |  |
| LM stenosis | 0.69 | 0.18-2.61 | .59 |  |  |  |  |
| Three-system disease | 1.51 | 0.40-5.68 | .55 |  |  |  |  |
| **Surgical factors** |  |  |  |  |  |  |  |
| Non-elective operation | 1.29 | 0.38-4.38 | .68 |  |  |  |  |
| RA to LAD (non-LIMA to LAD) | 1.44 | 0.18-11.72 | .73 |  |  |  |  |
| Non-BIMA | - | - | .98 |  |  |  |  |
| Stenosis of RA targeted coronary artery<70% | - | - | .98 |  |  |  |  |
| Arterial grafts<50% | - | - | .99 |  |  |  |  |
| Non-TAR | 1.59 | 0.50-5.06 | .44 |  |  |  |  |
| Incomplete revascularization | 0.31 | 0.04-2.44 | .26 |  |  |  |  |
| On-pump | - | - | .99 |  |  |  |  |
| **Postoperative Medication** |  |  |  |  |  |  |  |
| CCB for 6 months | 1.49 | 0.19-11.94 | >.99 |  |  |  |  |

Table S8. Univariate and Multivariate Logistic Regression Analysis of Risk Factors for Repeat Revascularization

|  | Univariate Estimates | | |  | Adjusted Estimates | | |
| --- | --- | --- | --- | --- | --- | --- | --- |
|  | OR | 95% CI | P |  | OR | 95% CI | P |
| **Preoperative factors** |  |  |  |  |  |  |  |
| Female | - | - | .98 |  |  |  |  |
| BMI ≥24 | 0.74 | 0.23-2.39 | .62 |  |  |  |  |
| BMI ≥28 | 2.39 | 0.70-8.18 | .17 |  |  |  |  |
| Aged 60 years or more | 0.66 | 0.18-2.49 | .54 |  |  |  |  |
| Aged 65 years or more | 0.47 | 0.06-3.71 | .47 |  |  |  |  |
| Aged 70 years or more | - | - | .98 |  |  |  |  |
| ACS at admission | 0.93 | 0.28-3.17 | .91 |  |  |  |  |
| MI at admission | 0.79 | 0.10-6.28 | .82 |  |  |  |  |
| Diabetes mellitus | 0.33 | 0.07-1.53 | .16 |  |  |  |  |
| Hypertension | 1.63 | 0.35-7.58 | .53 |  |  |  |  |
| COPD | - | - | .98 |  |  |  |  |
| Hyperlipemia | 0.32 | 0.08-1.20 | .09 |  | 0.29 | 0.08-1.08 | .07 |
| CKD | 1.11 | 0.14-8.96 | .92 |  |  |  |  |
| PVD | - | - | .97 |  |  |  |  |
| Prior MI | 0.89 | 0.26-3.00 | .85 |  |  |  |  |
| Prior stroke | - | - | .97 |  |  |  |  |
| Smoking history | 2.92 | 0.63-13.52 | .17 |  |  |  |  |
| Current smoking | 5.35 | 1.16-24.75 | .03 |  | 5.95 | 1.28-27.75 | .02 |
| NYHA classification III or IV | 1.08 | 0.32-3.70 | .90 |  |  |  |  |
| Pro-BNP>600pg/ml | - | - | .96 |  |  |  |  |
| LVESD>40mm | 0.95 | 0.12-7.63 | .96 |  |  |  |  |
| LVEDD>60mm | 2.22 | 0.27-18.42 | .46 |  |  |  |  |
| LVEF<40% | - | - | .99 |  |  |  |  |
| LVEF<50% | 0.84 | 0.10-6.67 | .87 |  |  |  |  |
| Anemia | 0.64 | 0.14-2.99 | .57 |  |  |  |  |
| Abnormal platelet count | 1.97 | 0.41-9.37 | .39 |  |  |  |  |
| LM stenosis | 2.61 | 0.78-8.75 | .12 |  |  |  |  |
| Three-system disease | 0.86 | 0.25-3.01 | .82 |  |  |  |  |
| **Surgical factors** |  |  |  |  |  |  |  |
| Non-elective operation | 0.23 | 0.03-1.77 | .16 |  |  |  |  |
| RA to LAD (non-LIMA to LAD) | 3.35 | 0.68-16.37 | .14 |  |  |  |  |
| Non-BIMA | - | - | .98 |  |  |  |  |
| Stenosis of RA targeted coronary artery<70% | 2.08 | 0.25-16.67 | .50 |  |  |  |  |
| Arterial grafts<50% | - | - | .99 |  |  |  |  |
| Non-TAR | 0.51 | 0.13-1.92 | .32 |  |  |  |  |
| Incomplete revascularization | 1.79 | 0.52-6.25 | .35 |  |  |  |  |
| On-pump | - | - | .99 |  |  |  |  |
| **Postoperative Medication** |  |  |  |  |  |  |  |
| CCB for 6 months | 1.65 | 0.21-13.07 | .97 |  |  |  |  |

Table S9. Univariate and Multivariate Analysis of Risk Factors for MACE-4 in the perioperative period

|  | Univariate Estimates | | |  | Adjusted Estimates | | |
| --- | --- | --- | --- | --- | --- | --- | --- |
|  | OR | 95% CI | P |  | OR | 95% CI | P |
| **Preoperative factors** |  |  |  |  |  |  |  |
| Female | 2.06 | 0.73-5.76 | .17 |  |  |  |  |
| BMI ≥24 | 0.62 | 0.30-1.31 | .21 |  |  |  |  |
| BMI ≥28 | 1.38 | 0.57-3.36 | .47 |  |  |  |  |
| Aged 60 years or more | 1.30 | 0.61-2.77 | .50 |  |  |  |  |
| Aged 65 years or more | 1.30 | 0.51-3.31 | .59 |  |  |  |  |
| Aged 70 years or more | 0.95 | 0.21-4.22 | .94 |  |  |  |  |
| ACS at admission | 1.16 | 0.52-2.60 | .72 |  |  |  |  |
| MI at admission | 4.37 | 1.84-10.37 | .001 |  | 3.75 | 1.46-9.63 | .006 |
| Diabetes mellitus | 1.67 | 0.80-3.49 | .18 |  |  |  |  |
| Hypertension | 0.65 | 0.29-1.43 | .29 |  |  |  |  |
| COPD | 1.30 | 0.16-10.89 | .81 |  |  |  |  |
| Hyperlipemia | 0.69 | 0.33-1.46 | .33 |  |  |  |  |
| CKD | 0.83 | 0.19-3.68 | .81 |  |  |  |  |
| PVD | 0.78 | 0.17-3.47 | .74 |  |  |  |  |
| Prior MI | 2.33 | 1.11-4.90 | .03 |  | 2.20 | 0.94-5.14 | .07 |
| Prior stroke | 0.96 | 0.28-3.33 | .95 |  |  |  |  |
| Smoking history | 0.89 | 0.42-1.90 | .76 |  |  |  |  |
| Current smoking | 1.46 | 0.69-3.07 | .32 |  |  |  |  |
| NYHA classification III or IV | 0.93 | 0.43-2.01 | .84 |  |  |  |  |
| Pro-BNP>600pg/ml | 2.09 | 0.83-5.28 | .12 |  |  |  |  |
| LVESD>40mm | 2.21 | 0.78-6.21 | .13 |  |  |  |  |
| LVEDD>60mm | 0.77 | 0.10-6.05 | .80 |  |  |  |  |
| LVEF<40% | - | - | .98 |  |  |  |  |
| LVEF<50% | 1.42 | 0.47-4.30 | .54 |  |  |  |  |
| Anemia | 0.94 | 0.39-2.27 | .90 |  |  |  |  |
| Abnormal platelet count | 1.98 | 0.71-5.54 | .19 |  |  |  |  |
| LM stenosis | 0.55 | 0.22-1.39 | .21 |  |  |  |  |
| Three-system disease | 2.42 | 0.90-6.56 | .08 |  | 1.93 | 0.68-5.46 | .22 |
| **Surgical factors** |  |  |  |  |  |  |  |
| Non-elective operation | 0.88 | 0.38-2.04 | .77 |  |  |  |  |
| RA to LAD (non-LIMA to LAD) | 1.79 | 0.50-6.44 | .38 |  |  |  |  |
| Non-BIMA | 1.19 | 0.15-9.09 | .87 |  |  |  |  |
| Stenosis of RA targeted coronary artery<70% | 2.74 | 0.73-10.28 | .13 |  |  |  |  |
| Arterial grafts<50% | 1.59 | 0.19-13.38 | .67 |  |  |  |  |
| Non-TAR | 1.32 | 0.63-2.78 | .48 |  |  |  |  |
| Incomplete revascularization | 2.08 | 0.94-4.55 | .07 |  | 2.17 | 0.93-5.26 | .07 |
| On-pump | 1.58 | 0.19-13.28 | .67 |  |  |  |  |
| **Postoperative Medication** |  |  |  |  |  |  |  |
| CCB for 6 months | 1.92 | 0.44-8.38 | .56 |  |  |  |  |

Table S10. Univariate and Multivariate Logistic Regression Analysis of Risk Factors for MACE-3 in the perioperative period

|  | Univariate Estimates | | |  | Adjusted Estimates | | |
| --- | --- | --- | --- | --- | --- | --- | --- |
|  | OR | 95% CI | P |  | OR | 95% CI | P |
| **Preoperative factors** |  |  |  |  |  |  |  |
| Female | 2.14 | 0.76-6.03 | .15 |  |  |  |  |
| BMI ≥24 | 0.68 | 0.32-1.44 | .31 |  |  |  |  |
| BMI ≥28 | 1.45 | 0.59-3.53 | .41 |  |  |  |  |
| Aged 60 years or more | 1.18 | 0.54-2.56 | .68 |  |  |  |  |
| Aged 65 years or more | 1.06 | 0.39-2.89 | .91 |  |  |  |  |
| Aged 70 years or more | 0.98 | 0.22-4.39 | .98 |  |  |  |  |
| ACS at admission | 1.10 | 0.49-2.48 | .82 |  |  |  |  |
| MI at admission | 4.60 | 1.93-10.96 | .001 |  | 2.57 | 0.95-6.96 | .06 |
| Diabetes mellitus | 1.79 | 0.84-3.78 | .13 |  |  |  |  |
| Hypertension | 0.62 | 0.28-1.37 | .23 |  |  |  |  |
| COPD | 1.37 | 0.16-11.46 | .77 |  |  |  |  |
| Hyperlipemia | 0.74 | 0.35-1.57 | .43 |  |  |  |  |
| CKD | 0.86 | 0.19-3.82 | .84 |  |  |  |  |
| PVD | 0.81 | 0.18-3.62 | .78 |  |  |  |  |
| Prior MI | 2.53 | 1.19-5.38 | .02 |  | 2.04 | 0.88-4.72 | .10 |
| Prior stroke | 1.00 | 0.29-3.47 | 1.00 |  |  |  |  |
| Smoking history | 0.84 | 0.39-1.80 | .65 |  |  |  |  |
| Current smoking | 1.37 | 0.65-2.91 | .41 |  |  |  |  |
| NYHA classification III or IV | 1.02 | 0.46-2.27 | .95 |  |  |  |  |
| Pro-BNP>600pg/ml | 2.20 | 0.87-5.58 | .10 |  | 1.37 | 0.50-3.74 | .55 |
| LVESD>40mm | 2.30 | 0.82-6.50 | .12 |  |  |  |  |
| LVEDD>60mm | 0.80 | 0.10-6.29 | .83 |  |  |  |  |
| LVEF<40% | - | - | .98 |  |  |  |  |
| LVEF<50% | 1.48 | 0.48-4.50 | .49 |  |  |  |  |
| Anemia | 0.99 | 0.41-2.39 | .98 |  |  |  |  |
| Abnormal platelet count | 2.07 | 0.74-5.79 | .17 |  |  |  |  |
| LM stenosis | 0.58 | 0.23-1.47 | .25 |  |  |  |  |
| Three-system disease | 2.31 | 0.85-6.27 | .10 |  |  |  |  |
| **Surgical factors** |  |  |  |  |  |  |  |
| Non-elective operation | 0.92 | 0.40-2.15 | .86 |  |  |  |  |
| RA to LAD (non-LIMA to LAD) | 1.13 | 0.25-5.09 | .88 |  |  |  |  |
| Non-BIMA | 1.14 | 0.15-9.08 | .89 |  |  |  |  |
| Stenosis of RA targeted coronary artery<70% | 2.86 | 0.76-10.69 | .12 |  |  |  |  |
| Arterial grafts<50% | 1.64 | 0.19-13.79 | .65 |  |  |  |  |
| Non-TAR | 1.39 | 0.66-2.94 | .38 |  |  |  |  |
| Incomplete revascularization | 1.85 | 0.83-4.17 | .13 |  |  |  |  |
| On-pump | 1.64 | 0.20-13.80 | .65 |  |  |  |  |
| **Postoperative Medication** |  |  |  |  |  |  |  |
| CCB for 6 months | 1.84 | 0.42-8.04 | .61 |  |  |  |  |

Table S11. Univariate and Multivariate Logistic Regression Analysis of Risk Factors for All-cause death in the perioperative period

|  | Univariate Estimates | | |  | Adjusted Estimates | | |
| --- | --- | --- | --- | --- | --- | --- | --- |
|  | OR | 95% CI | P |  | OR | 95% CI | P |
| **Preoperative factors** |  |  |  |  |  |  |  |
| Female | 32.42 | 3.27-321.06 | .003 |  | - | - | .16 |
| BMI ≥24 | 0.53 | 0.07-3.82 | .53 |  |  |  |  |
| BMI ≥28 | 1.54 | 0.16-15.08 | .71 |  |  |  |  |
| Aged 60 years or more | 6.15 | 0.63-59.75 | .12 |  |  |  |  |
| Aged 65 years or more | 5.42 | 0.75-39.27 | .09 |  | - | - | .21 |
| Aged 70 years or more | 4.75 | 0.48-47.41 | .18 |  |  |  |  |
| ACS at admission | - | - | .96 |  |  |  |  |
| MI at admission | 9.17 | 1.25-67.05 | .03 |  | - | - | .74 |
| Diabetes mellitus | - | - | .96 |  |  |  |  |
| Hypertension | 0.32 | 0.04-2.28 | .25 |  |  |  |  |
| COPD | 16.32 | 1.34-199.02 | .03 |  | - | - | .15 |
| Hyperlipemia | 0.33 | 0.03-3.16 | .33 |  |  |  |  |
| CKD | - | - | .97 |  |  |  |  |
| PVD | - | - | .97 |  |  |  |  |
| Prior MI | 1.79 | 0.25-12.88 | .56 |  |  |  |  |
| Prior stroke | - | - | .97 |  |  |  |  |
| Smoking history | 0.19 | 0.02-1.80 | .15 |  |  |  |  |
| Current smoking | 0.34 | 0.03-3.27 | .35 |  |  |  |  |
| NYHA classification III or IV | 0.18 | 0.02-1.69 | .13 |  |  |  |  |
| Pro-BNP>600pg/ml | 19.30 | 1.96-190.47 | .01 |  | - | - | .20 |
| LVESD>40mm | - | - | .97 |  |  |  |  |
| LVEDD>60mm | - | - | .98 |  |  |  |  |
| LVEF<40% | - | - | .98 |  |  |  |  |
| LVEF<50% | 3.14 | 0.32-31.03 | .33 |  |  |  |  |
| Anemia | 1.09 | 0.11-10.57 | .94 |  |  |  |  |
| Abnormal platelet count | 10.09 | 1.38-73.99 | .02 |  | - | - | .15 |
| LM stenosis | 2.12 | 0.29-15.26 | .46 |  |  |  |  |
| Three-system disease | 1.49 | 0.15-14.52 | .73 |  |  |  |  |
| Surgical factors |  |  |  |  |  |  |  |
| Non-elective operation | 2.59 | 0.36-18.62 | .35 |  |  |  |  |
| RA to LAD (non-LIMA to LAD) | 5.42 | 0.54-54.45 | .15 |  |  |  |  |
| Non-BIMA | - | - | .98 |  |  |  |  |
| Stenosis of RA targeted coronary artery<70% | 0.99 | 0.97-1.01 | >.99 |  |  |  |  |
| Arterial grafts<50% | 16.67 | 1.59-175.07 | .02 |  | - | - | .93 |
| Non-TAR | 1.56 | 0.22-11.23 | .66 |  |  |  |  |
| Incomplete revascularization | 1.16 | 0.12-11.29 | .89 |  |  |  |  |
| On-pump | 17.10 | 1.58-185.37 | .02 |  | - | - | .81 |
| **Postoperative Medication** |  |  |  |  |  |  |  |
| CCB for 6 months | - | - | - |  |  |  |  |

Table S12. Univariate and Multivariate Logistic Regression Analysis of Risk Factors for CV-death in the perioperative period

|  | Univariate Estimates | | |  | Adjusted Estimates | | |
| --- | --- | --- | --- | --- | --- | --- | --- |
|  | OR | 95% CI | P |  | OR | 95% CI | P |
| **Preoperative factors** |  |  |  |  |  |  |  |
| Female | 32.42 | 3.27-321.06 | .003 |  | - | - | .16 |
| BMI ≥24 | 0.53 | 0.07-3.82 | .53 |  |  |  |  |
| BMI ≥28 | 1.54 | 0.16-15.08 | .71 |  |  |  |  |
| Aged 60 years or more | 6.15 | 0.63-59.75 | .12 |  |  |  |  |
| Aged 65 years or more | 5.42 | 0.75-39.27 | .09 |  | - | - | .21 |
| Aged 70 years or more | 4.75 | 0.48-47.41 | .18 |  |  |  |  |
| ACS at admission | - | - | .96 |  |  |  |  |
| MI at admission | 9.17 | 1.25-67.05 | .03 |  | - | - | .74 |
| Diabetes mellitus | - | - | .96 |  |  |  |  |
| Hypertension | 0.32 | 0.04-2.28 | .25 |  |  |  |  |
| COPD | 16.32 | 1.34-199.02 | .03 |  | - | - | .15 |
| Hyperlipemia | 0.33 | 0.03-3.16 | .33 |  |  |  |  |
| CKD | - | - | .97 |  |  |  |  |
| PVD | - | - | .97 |  |  |  |  |
| Prior MI | 1.79 | 0.25-12.88 | .56 |  |  |  |  |
| Prior stroke | - | - | .97 |  |  |  |  |
| Smoking history | 0.19 | 0.02-1.80 | .15 |  |  |  |  |
| Current smoking | 0.34 | 0.03-3.27 | .35 |  |  |  |  |
| NYHA classification III or IV | 0.18 | 0.02-1.69 | .13 |  |  |  |  |
| Pro-BNP>600pg/ml | 19.30 | 1.96-190.47 | .01 |  | - | - | .20 |
| LVESD>40mm | - | - | .97 |  |  |  |  |
| LVEDD>60mm | - | - | .98 |  |  |  |  |
| LVEF<40% | - | - | .98 |  |  |  |  |
| LVEF<50% | 3.14 | 0.32-31.03 | .33 |  |  |  |  |
| Anemia | 1.09 | 0.11-10.57 | .94 |  |  |  |  |
| Abnormal platelet count | 10.09 | 1.38-73.99 | .02 |  | - | - | .15 |
| LM stenosis | 2.12 | 0.29-15.26 | .46 |  |  |  |  |
| Three-system disease | 1.49 | 0.15-14.52 | .73 |  |  |  |  |
| **Surgical factors** |  |  |  |  |  |  |  |
| Non-elective operation | 2.59 | 0.36-18.62 | .35 |  |  |  |  |
| RA to LAD (non-LIMA to LAD) | 5.42 | 0.54-54.45 | .15 |  |  |  |  |
| Non-BIMA | - | - | .98 |  |  |  |  |
| Stenosis of RA targeted coronary artery<70% | 0.99 | 0.97-1.01 | >.99 |  |  |  |  |
| Arterial grafts<50% | 16.67 | 1.59-174.70 | .02 |  | - | - | .93 |
| Non-TAR | 1.56 | 0.22-11.23 | .66 |  |  |  |  |
| Incomplete revascularization | 1.16 | 0.12-11.29 | .89 |  |  |  |  |
| On-pump | 17.10 | 1.58-185.37 | .02 |  | - | - | .81 |
| **Postoperative Medication** |  |  |  |  |  |  |  |
| CCB for 6 months | - | - | - |  |  |  |  |

Table S13. Univariate and Multivariate Logistic Regression Analysis of Risk Factors for MI in the perioperative period

|  | Univariate Estimates | | |  | Adjusted Estimates | | |
| --- | --- | --- | --- | --- | --- | --- | --- |
|  | OR | 95% CI | P |  | OR | 95% CI | P |
| **Preoperative factors** |  |  |  |  |  |  |  |
| Female | 1.73 | 0.56-5.33 | .34 |  |  |  |  |
| BMI ≥24 | 0.59 | 0.27-1.29 | .19 |  |  |  |  |
| BMI ≥28 | 1.60 | 0.65-3.93 | .31 |  |  |  |  |
| Aged 60 years or more | 1.13 | 0.50-2.52 | .77 |  |  |  |  |
| Aged 65 years or more | 0.87 | 0.29-2.61 | .80 |  |  |  |  |
| Aged 70 years or more | 1.07 | 0.24-4.78 | .93 |  |  |  |  |
| ACS at admission | 0.99 | 0.43-2.25 | .98 |  |  |  |  |
| MI at admission | 5.11 | 2.12-12.32 | <.001 |  | 3.90 | 1.50-10.32 | .006 |
| Diabetes mellitus | 1.78 | 0.82-3.86 | .14 |  |  |  |  |
| Hypertension | 0.66 | 0.29-1.50 | .32 |  |  |  |  |
| COPD | - | - | .98 |  |  |  |  |
| Hyperlipemia | 0.72 | 0.33-1.58 | .42 |  |  |  |  |
| CKD | 0.93 | 0.21-4.16 | .93 |  |  |  |  |
| PVD | 0.88 | 0.20-3.95 | .87 |  |  |  |  |
| Prior MI | 2.56 | 1.17-5.60 | .02 ^a^ |  | 2.21 | 0.86-5.70 | .10 |
| Prior stroke | 1.09 | 0.31-3.79 | .90 |  |  |  |  |
| Smoking history | 0.87 | 0.39-1.91 | .73 |  |  |  |  |
| Current smoking | 1.40 | 0.64-3.04 | .40 |  |  |  |  |
| NYHA classification III or IV | 1.09 | 0.47-2.50 | .85 |  |  |  |  |
| Pro-BNP>600pg/ml | 1.93 | 0.72-5.16 | .19 |  |  |  |  |
| LVESD>40mm | 2.52 | 0.89-7.16 | .08 ^a^ |  | 1.40 | 0.44-4.43 | .57 |
| LVEDD>60mm | 0.86 | 0.11-6.81 | .89 |  |  |  |  |
| LVEF<40% | - | - | .98 |  |  |  |  |
| LVEF<50% | 1.61 | 0.53-4.93 | .41 |  |  |  |  |
| Anemia | 1.09 | 0.45-2.66 | .85 |  |  |  |  |
| Abnormal platelet count | 2.26 | 0.80-6.38 | .12 |  |  |  |  |
| LM stenosis | 0.64 | 0.25-1.66 | .36 |  |  |  |  |
| Three-system disease | 2.08 | 0.76-5.69 | .16 |  |  |  |  |
| **Surgical factors** |  |  |  |  |  |  |  |
| Non-elective operation | 0.84 | 0.35-2.04 | .70 |  |  |  |  |
| RA to LAD (non-LIMA to LAD) | 1.22 | 0.27-5.54 | .79 |  |  |  |  |
| Non-BIMA | 1.06 | 0.13-8.33 | .95 |  |  |  |  |
| Stenosis of RA targeted coronary artery<70% | 3.13 | 0.83-11.72 | .09 ^a^ |  | 2.63 | 0.65-10.73 | .18 |
| Arterial grafts<50% | 1.79 | 0.21-15.19 | .60 |  |  |  |  |
| Non-TAR | 1.61 | 0.75-3.45 | .23 |  |  |  |  |
| Incomplete revascularization | 1.72 | 0.75-3.95 | .20 |  |  |  |  |
| On-pump | 1.77 | 0.21-14.94 | .60 |  |  |  |  |
| **Postoperative Medication** |  |  |  |  |  |  |  |
| CCB for 6 months | 1.74 | 0.40-7.70 | .66 |  |  |  |  |

Table S14. Univariate and Multivariate Logistic Regression Analysis of Risk Factors for Stroke in the perioperative period

|  | Univariate Estimates | | |  | Adjusted Estimates | | |
| --- | --- | --- | --- | --- | --- | --- | --- |
|  | OR | 95% CI | P |  | OR | 95% CI | P |
| **Preoperative factors** |  |  |  |  |  |  |  |
| Female | 10.15 | 0.62-166.06 | .10 |  |  |  |  |
| BMI ≥24 | - | - | .95 |  |  |  |  |
| BMI ≥28 | - | - | .97 |  |  |  |  |
| Aged 60 years or more | 2.02 | 0.13-32.51 | .62 |  |  |  |  |
| Aged 65 years or more | 5.34 | 0.33-86.67 | .24 |  |  |  |  |
| Aged 70 years or more | - | - | .98 |  |  |  |  |
| ACS at admission | - | - | .96 |  |  |  |  |
| MI at admission | - | - | .98 |  |  |  |  |
| Diabetes mellitus | 1.71 | 0.11-27.49 | .71 |  |  |  |  |
| Hypertension | 0.32 | 0.02-5.15 | .42 |  |  |  |  |
| COPD | 32.75 | 1.88-571.68 | .02 |  | - | - | - |
| Hyperlipemia | 0.99 | 0.06-15.93 | .99 |  |  |  |  |
| CKD | - | - | .98 |  |  |  |  |
| PVD | - | - | .98 |  |  |  |  |
| Prior MI | 1.79 | 0.11-28.82 | .68 |  |  |  |  |
| Prior stroke | - | - | .98 |  |  |  |  |
| Smoking history | 0.57 | 0.04-9.12 | .69 |  |  |  |  |
| Current smoking | 1.02 | 0.06-16.46 | .99 |  |  |  |  |
| NYHA classification III or IV | 0.53 | 0.03-8.33 | .66 |  |  |  |  |
| Pro-BNP>600pg/ml | 6.10 | 0.37-99.60 | .20 |  |  |  |  |
| LVESD>40mm | - | - | .98 |  |  |  |  |
| LVEDD>60mm | - | - | .99 |  |  |  |  |
| LVEF<40% | - | - | .99 |  |  |  |  |
| LVEF<50% | - | - | .98 |  |  |  |  |
| Anemia | - | - | .96 |  |  |  |  |
| Abnormal platelet count | - | - | .98 |  |  |  |  |
| LM stenosis | - | - | .96 |  |  |  |  |
| Three-system disease | - | - | .96 |  |  |  |  |
| **Surgical factors** |  |  |  |  |  |  |  |
| Non-elective operation | 2.57 | 0.16-41.52 | .51 |  |  |  |  |
| RA to LAD (non-LIMA to LAD) | - | - | .98 |  |  |  |  |
| Non-BIMA | - | - | .99 |  |  |  |  |
| Stenosis of RA targeted coronary artery<70% | 0.99 | 0.98-1.00 | >.99 |  |  |  |  |
| Arterial grafts<50% | - | - | .99 |  |  |  |  |
| Non-TAR | - | - | .95 |  |  |  |  |
| Incomplete revascularization | 3.57 | 0.22-58.55 | .38 |  |  |  |  |
| On-pump | - | - | .99 |  |  |  |  |
| **Postoperative Medication** |  |  |  |  |  |  |  |
| CCB for 6 months | 1.00 | 1.00-1.01 | >.99 |  |  |  |  |

Table S15. Univariate and Multivariate Logistic Regression Analysis of Risk Factors for Repeat revascularization in the perioperative period

|  | Univariate estimates | | |
| --- | --- | --- | --- |
|  | OR | 95% CI | P |
| **Preoperative factors** |  |  |  |
| Female | - | - | .98 |
| BMI ≥24 | - | - | .95 |
| BMI ≥28 | - | - | .97 |
| Aged 60 years or more | - | - | .95 |
| Aged 65 years or more | - | - | .96 |
| Aged 70 years or more | - | - | .98 |
| ACS at admission | - | - | .95 |
| MI at admission | - | - | .97 |
| Diabetes mellitus | - | - | .95 |
| Hypertension | - |  | .96 |
| COPD | - | - | .99 |
| Hyperlipemia | - |  | .96 |
| CKD | - | - | .98 |
| PVD | - | - | .98 |
| Prior MI | - |  | .95 |
| Prior stroke | - | - | .97 |
| Smoking history | - | - | .95 |
| Current smoking | - | - | .96 |
| NYHA classification III or IV | - | - | .95 |
| Pro-BNP>600pg/ml | - | - | .97 |
| LVESD>40mm | - | - | .98 |
| LVEDD>60mm | - |  | .98 |
| LVEF<40% | - | - | .99 |
| LVEF<50% | - | - | .98 |
| Anemia | - | - | .96 |
| Abnormal platelet count | - | - | .98 |
| LM stenosis | - | - | .95 |
| Three-system disease | - | - | .95 |
| **Surgical factors** |  |  |  |
| Non-elective operation | - | - | .96 |
| RA to LAD (non-LIMA to LAD) | - | - | .97 |
| Non-BIMA | - | - | .98 |
| Stenosis of RA targeted coronary artery<70% | 1.00 | 0.99-1.01 | >.99 |
| Arterial grafts<50% | - | - | .99 |
| Non-TAR | - | - | .97 |
| Incomplete revascularization | - | - | .96 |
| On-pump | - | - | .99 |
| **Postoperative Medication** |  |  |  |
| CCB for 6 months | 1.00 | 1.00-1.01 | >.99 |

Table S16. Univariate and Multivariate Logistic Regression Analysis of Risk Factors for MACE-3 in the early period

|  | Univariate Estimates | | |  | Adjusted Estimates | | |
| --- | --- | --- | --- | --- | --- | --- | --- |
|  | OR | 95% CI | P |  | OR | 95% CI | P |
| **Preoperative factors** |  |  |  |  |  |  |  |
| Female | 0.26 | 0.03-1.94 | .19 |  |  |  |  |
| BMI ≥24 | 1.19 | 0.58-2.44 | .64 |  |  |  |  |
| BMI ≥28 | 1.51 | 0.67-3.37 | .32 |  |  |  |  |
| Aged 60 years or more | 0.78 | 0.37-1.63 | .51 |  |  |  |  |
| Aged 65 years or more | 1.18 | 0.49-2.84 | .71 |  |  |  |  |
| Aged 70 years or more | 2.31 | 0.81-6.63 | .12 |  |  |  |  |
| ACS at admission | 0.94 | 0.46-1.90 | .86 |  |  |  |  |
| MI at admission | 1.70 | 0.61-4.74 | .31 |  |  |  |  |
| Diabetes mellitus | 0.88 | 0.43-1.78 | .72 |  |  |  |  |
| Hypertension | 1.46 | 0.62-3.46 | .39 |  |  |  |  |
| COPD | 5.19 | 1.17-23.01 | .03 |  | 3.19 | 0.47-21.50 | .23 |
| Hyperlipemia | 0.85 | 0.43-1.68 | .64 |  |  |  |  |
| CKD | 1.01 | 0.29-3.52 | .99 |  |  |  |  |
| PVD | 2.13 | 0.73-6.16 | .16 |  |  |  |  |
| Prior MI | 3.63 | 1.82-7.25 | <.001 |  | 3.48 | 1.10-10.99 | .03 |
| Prior stroke | 1.77 | 0.68-4.60 | .24 |  |  |  |  |
| Smoking history | 1.72 | 0.81-3.66 | .16 |  |  |  |  |
| Current smoking | 1.59 | 0.81-3.13 | .18 |  |  |  |  |
| NYHA classification III or IV | 0.85 | 0.43-1.69 | .63 |  |  |  |  |
| Pro-BNP>600pg/ml | 2.67 | 1.03-6.91 | .04 |  | 0.84 | 0.18-4.04 | .83 |
| LVESD>40mm | 3.80 | 1.54-9.41 | .004 |  | 2.20 | 0.17-28.48 | .54 |
| LVEDD>60mm | 3.30 | 0.98-11.09 | .05 |  | 0.40 | 0.03-5.73 | .50 |
| LVEF<40% | 4.92 | 1.13-21.44 | .03 |  | 1.20 | 0.06-26.17 | .91 |
| LVEF<50% | 2.97 | 1.23-7.17 | .02 |  | 0.96 | 0.12-7.88 | .97 |
| Anemia | 1.32 | 0.63-2.79 | .47 |  |  |  |  |
| Abnormal platelet count | 0.53 | 0.12-2.30 | .40 |  |  |  |  |
| LM stenosis | 0.30 | 0.11-0.81 | .02 |  | 0.22 | 0.04-1.07 | .06 |
| Three-system disease | 1.16 | 0.55-2.48 | .69 |  |  |  |  |
| **Surgical factors** |  |  |  |  |  |  |  |
| Non-elective operation | 1.70 | 0.85-3.40 | .14 |  |  |  |  |
| RA to LAD (non-LIMA to LAD) | 2.16 | 0.68-6.89 | .19 |  |  |  |  |
| Non-BIMA | 1.59 | 0.20-12.62 | .67 |  |  |  |  |
| Stenosis of RA targeted coronary artery<70% | 2.50 | 0.65-9.56 | .18 |  |  |  |  |
| Arterial grafts<50% | 3.23 | 0.60-17.38 | .17 |  |  |  |  |
| Non-TAR | 1.61 | 0.82-3.13 | .17 |  |  |  |  |
| Incomplete revascularization | 1.12 | 0.51-2.49 | .77 |  |  |  |  |
| On-pump | 3.20 | 0.60-17.09 | .17 |  |  |  |  |
| **Postoperative Medication** |  |  |  |  |  |  |  |
| CCB for 6 months | 1.01 | 0.37-2.72 | >.99 |  |  |  |  |

Table S17. Univariate and Multivariate Logistic Regression Analysis of Risk Factors for All-cause death in the early period

|  | Univariate Estimates | | |  | Adjusted Estimates | | |
| --- | --- | --- | --- | --- | --- | --- | --- |
|  | OR | 95% CI | p |  | OR | 95% CI | P |
| **Preoperative factors** |  |  |  |  |  |  |  |
| Female | 2.50 | 0.52-12.11 | .26 |  |  |  |  |
| BMI ≥24 | 0.93 | 0.27-3.23 | .91 |  |  |  |  |
| BMI ≥28 | 1.03 | 0.22-4.88 | .97 |  |  |  |  |
| Aged 60 years or more | 1.74 | 0.52-5.82 | .37 |  |  |  |  |
| Aged 65 years or more | 2.09 | 0.54-8.13 | .29 |  |  |  |  |
| Aged 70 years or more | 3.36 | 0.68-16.52 | .14 |  |  |  |  |
| ACS at admission | 0.83 | 0.24-2.88 | .77 |  |  |  |  |
| MI at admission | - | - | .96 |  |  |  |  |
| Diabetes mellitus | 3.03 | 0.87-10.54 | .08 |  | 2.54 | 0.61-10.51 | .20 |
| Hypertension | 3.25 | 0.41-25.72 | .27 |  |  |  |  |
| COPD | - | - | .98 |  |  |  |  |
| Hyperlipemia | 0.81 | 0.24-2.70 | .73 |  |  |  |  |
| CKD | 4.95 | 1.24-19.83 | .02 |  | 5.23 | 1.08-25.34 | .04 |
| PVD | 1.86 | 0.22-16.10 | .57 |  |  |  |  |
| Prior MI | 1.51 | 0.45-5.06 | .50 |  |  |  |  |
| Prior stroke | 2.03 | 0.42-9.78 | .38 |  |  |  |  |
| Smoking history | 1.50 | 0.39-5.77 | .55 |  |  |  |  |
| Current smoking | 1.22 | 0.37-4.07 | .75 |  |  |  |  |
| NYHA classification III or IV | 0.29 | 0.08-1.01 | .05 |  | 0.24 | 0.06-1.08 | .06 |
| Pro-BNP>600pg/ml | 2.66 | 0.50-14.26 | .25 |  |  |  |  |
| LVESD>40mm | 4.19 | 1.05-16.66 | .04 |  | 0.26 | 0.01-8.37 | .45 |
| LVEDD>60mm | 5.81 | 1.14-29.64 | .03 |  | 16.02 | 0.40-636.23 | .14 |
| LVEF<40% | 12.89 | 2.28-72.84 | .004 |  | 1.87 | 0.08-41.41 | .69 |
| LVEF<50% | 2.16 | 0.45-10.43 | .34 |  |  |  |  |
| Anemia | 1.90 | 0.54-6.66 | .31 |  |  |  |  |
| Abnormal platelet count | - | - | .96 |  |  |  |  |
| LM stenosis | 0.91 | 0.23-3.58 | .89 |  |  |  |  |
| Three-system disease | 0.74 | 0.20-2.68 | .65 |  |  |  |  |
| **Surgical factors** |  |  |  |  |  |  |  |
| Non-elective operation | 2.22 | 0.66-7.43 | .20 |  |  |  |  |
| RA to LAD (non-LIMA to LAD) | 1.85 | 0.22-15.35 | .57 |  |  |  |  |
| Non-BIMA | - | - | .98 |  |  |  |  |
| Stenosis of RA targeted coronary artery<70% | 2.27 | 0.27-19.01 | .45 |  |  |  |  |
| Arterial grafts<50% | - | - | .98 |  |  |  |  |
| Non-TAR | 0.34 | 0.07-1.59 | .17 |  |  |  |  |
| Incomplete revascularization | 0.78 | 0.16-3.70 | .74 |  |  |  |  |
| On-pump | 15.56 | 2.65-91.17 | .002 |  | 58.22 | 5.26-643.92 | <.001 |
| **Postoperative Medication** |  |  |  |  |  |  |  |
| CCB for 6 months | 1.34 | 0.17-10.82 | >.99 |  |  |  |  |

Table S18. Univariate and Multivariate Logistic Regression Analysis of Risk Factors for MI in the early period

|  | Univariate Estimates | | |  | Adjusted Estimates | | |
| --- | --- | --- | --- | --- | --- | --- | --- |
|  | OR | 95% CI | P |  | OR | 95% CI | P |
| **Preoperative factors** |  |  |  |  |  |  |  |
| Female | 0.29 | 0.04-2.21 | .23 |  |  |  |  |
| BMI ≥24 | 1.08 | 0.51-2.30 | .84 |  |  |  |  |
| BMI ≥28 | 1.28 | 0.53-3.09 | .59 |  |  |  |  |
| Aged 60 years or more | 0.83 | 0.38-1.80 | .64 |  |  |  |  |
| Aged 65 years or more | 1.40 | 0.58-3.41 | .45 |  |  |  |  |
| Aged 70 years or more | 2.78 | 0.96-8.03 | .06 |  | 6.2 | 0.96-40.13 | .06 |
| ACS at admission | 0.98 | 0.46-2.08 | .95 |  |  |  |  |
| MI at admission | 2.04 | 0.72-5.75 | .18 |  |  |  |  |
| Diabetes mellitus | 0.84 | 0.39-1.78 | .64 |  |  |  |  |
| Hypertension | 1.22 | 0.51-2.92 | .66 |  |  |  |  |
| COPD | 5.00 | 1.17-21.39 | .03 |  | 2.72 | 0.40-18.65 | .31 |
| Hyperlipemia | 0.90 | 0.44-1.84 | .77 |  |  |  |  |
| CKD | 0.74 | 0.17-3.28 | .69 |  |  |  |  |
| PVD | 2.43 | 0.83-7.08 | .11 |  |  |  |  |
| Prior MI | 4.11 | 1.95-8.65 | <.001 |  | 7.79 | 1.76-34.41 | .007 |
| Prior stroke | 1.66 | 0.60-4.61 | .33 |  |  |  |  |
| Smoking history | 1.63 | 0.74-3.62 | .23 |  |  |  |  |
| Current smoking | 1.37 | 0.67-2.79 | .39 |  |  |  |  |
| NYHA classification III or IV | 0.86 | 0.41-1.79 | .69 |  |  |  |  |
| Pro-BNP>600pg/ml | 2.55 | 0.93-7.00 | .07 |  | 0.58 | 0.12-2.89 | .51 |
| LVESD>40mm | 3.71 | 1.44-9.56 | .007 |  | 0.98 | 0.11-8.40 | .99 |
| LVEDD>60mm | 2.60 | 0.69-9.81 | .16 |  |  |  |  |
| LVEF<40% | 3.14 | 0.61-16.19 | .17 |  |  |  |  |
| LVEF<50% | 2.93 | 1.16-7.39 | .02 |  | 1.46 | 0.18-11.77 | .72 |
| Anemia | 1.42 | 0.65-3.10 | .38 |  |  |  |  |
| Abnormal platelet count | 0.63 | 0.14-2.75 | .53 |  |  |  |  |
| LM stenosis | 0.20 | 0.06-0.66 | .008 |  | 0.15 | 0.02-1.24 | .08 |
| Three-system disease | 1.56 | 0.67-3.61 | .30 |  |  |  |  |
| **Surgical factors** |  |  |  |  |  |  |  |
| Non-elective operation | 1.65 | 0.79-3.45 | .18 |  |  |  |  |
| RA to LAD (non-LIMA to LAD) | 2.51 | 0.78-8.07 | .12 |  |  |  |  |
| Non-BIMA | 1.33 | 0.17-10.54 | .78 |  |  |  |  |
| Stenosis of RA targeted coronary artery<70% | 1.69 | 0.36-8.33 | .50 |  |  |  |  |
| Arterial grafts<50% | 3.85 | 0.70-21.01 | .12 |  |  |  |  |
| Non-TAR | 1.92 | 0.95-3.88 | .07 |  | 0.69 | 0.21-2.27 | .54 |
| Incomplete revascularization | 1.37 | 0.61-3.10 | .45 |  |  |  |  |
| On-pump | 3.79 | 0.71-20.33 | .12 |  |  |  |  |
| **Postoperative Medication** |  |  |  |  |  |  |  |
| CCB for 6 months | 0.84 | 0.31-2.30 | .95 |  |  |  |  |

Table S19. Univariate and Multivariate Logistic Regression Analysis of Risk Factors for MACE-4 in the early period

|  | Univariate Estimates | | |  | Adjusted Estimates | | |
| --- | --- | --- | --- | --- | --- | --- | --- |
|  | OR | 95% CI | P |  | OR | 95% CI | P |
| **Preoperative factors** |  |  |  |  |  |  |  |
| Female | 0.66 | 0.19-2.28 | .51 |  |  |  |  |
| BMI ≥24 | 1.19 | 0.62-2.29 | .60 |  |  |  |  |
| BMI ≥28 | 1.46 | 0.70-3.06 | .32 |  |  |  |  |
| Aged 60 years or more | 1.00 | 0.52-1.90 | .99 |  |  |  |  |
| Aged 65 years or more | 1.26 | 0.57-2.77 | .57 |  |  |  |  |
| Aged 70 years or more | 2.24 | 0.84-6.00 | .11 |  |  |  |  |
| ACS at admission | 0.87 | 0.46-1.66 | .68 |  |  |  |  |
| MI at admission | 1.26 | 0.46-3.48 | .66 |  |  |  |  |
| Diabetes mellitus | 0.94 | 0.50-1.77 | .84 |  |  |  |  |
| Hypertension | 2.02 | 0.87-4.69 | .10 |  |  |  |  |
| COPD | 3.76 | 0.86-16.50 | .08 |  | 3.03 | 0.63-14.58 | .17 |
| Hyperlipemia | 0.95 | 0.52-1.76 | .88 |  |  |  |  |
| CKD | 1.08 | 0.36-3.29 | .89 |  |  |  |  |
| PVD | 1.71 | 0.60-4.89 | .32 |  |  |  |  |
| Prior MI | 2.51 | 1.36-4.64 | .003 |  | 1.83 | 0.78-4.26 | .16 |
| Prior stroke | 1.62 | 0.66-3.96 | .29 |  |  |  |  |
| Smoking history | 1.48 | 0.76-2.88 | .25 |  |  |  |  |
| Current smoking | 1.35 | 0.74-2.48 | .33 |  |  |  |  |
| NYHA classification III or IV | 0.74 | 0.40-1.37 | .34 |  |  |  |  |
| Pro-BNP>600pg/ml | 1.98 | 0.78-5.01 | .15 |  |  |  |  |
| LVESD>40mm | 3.38 | 1.42-8.03 | .006 |  | 1.75 | 0.24-12.76 | .58 |
| LVEDD>60mm | 3.52 | 1.13-11.00 | .03 |  | 0.83 | 0.09-7.28 | .87 |
| LVEF<40% | 3.70 | 0.86-16.03 | .08 |  | 1.09 | 0.10-12.10 | .94 |
| LVEF<50% | 2.60 | 1.12-6.02 | .03 |  | 0.96 | 0.17-5.32 | .96 |
| Anemia | 1.20 | 0.60-2.40 | .60 |  |  |  |  |
| Abnormal platelet count | 0.64 | 0.19-2.18 | .47 |  |  |  |  |
| LM stenosis | 0.45 | 0.21-0.98 | .05 |  | 0.39 | 0.14-1.08 | .07 |
| Three-system disease | 1.06 | 0.54-2.08 | .86 |  |  |  |  |
| **Surgical factors** |  |  |  |  |  |  |  |
| Non-elective operation | 1.42 | 0.75-2.70 | .29 |  |  |  |  |
| RA to LAD (non-LIMA to LAD) | 2.36 | 0.80-6.97 | .12 |  |  |  |  |
| Non-BIMA | 2.08 | 0.26-16.49 | .49 |  |  |  |  |
| Stenosis of RA targeted coronary artery<70% | 2.77 | 0.82-9.09 | .10 |  |  |  |  |
| Arterial grafts<50% | 2.44 | 0.46-13.03 | .30 |  |  |  |  |
| Non-TAR | 1.11 | 0.60-2.04 | .74 |  |  |  |  |
| Incomplete revascularization | 1.43 | 0.71-2.86 | .31 |  |  |  |  |
| On-pump | 4.66 | 1.01-21.52 | .05 |  | 5.44 | 0.61-48.79 | .13 |
| **Postoperative Medication** |  |  |  |  |  |  |  |
| CCB for 6 months | 1.04 | 0.42-2.60 | .93 |  |  |  |  |

Table S20. Univariate and Multivariate Logistic Regression Analysis of Risk Factors for CV-death in the early period

|  | Univariate Estimates | | |  | Adjusted Estimates | | |
| --- | --- | --- | --- | --- | --- | --- | --- |
|  | OR | 95% CI | P |  | OR | 95% CI | P |
| **Preoperative factors** |  |  |  |  |  |  |  |
| Female | - | - | .97 |  |  |  |  |
| BMI ≥24 | 1.06 | 0.19-5.89 | .94 |  |  |  |  |
| BMI ≥28 | 2.36 | 0.42-13.15 | .33 |  |  |  |  |
| Aged 60 years or more | 1.03 | 0.19-5.68 | .98 |  |  |  |  |
| Aged 65 years or more | 1.09 | 0.12-9.47 | .94 |  |  |  |  |
| Aged 70 years or more | 2.93 | 0.33-26.14 | .34 |  |  |  |  |
| ACS at admission | 0.47 | 0.09-2.36 | .36 |  |  |  |  |
| MI at admission | - | - | .97 |  |  |  |  |
| Diabetes mellitus | 3.41 | 0.62-18.89 | .16 |  |  |  |  |
| Hypertension | 1.59 | 0.18-13.82 | .67 |  |  |  |  |
| COPD | - | - | .99 |  |  |  |  |
| Hyperlipemia | 0.19 | 0.02-1.65 | .13 |  |  |  |  |
| CKD | 6.42 | 1.12-36.73 | .04 |  | 3.43 | 0.26-45.13 | .35 |
| PVD | 3.77 | 0.38-37.60 | .26 |  |  |  |  |
| Prior MI | 3.67 | 0.66-20.31 | .14 |  |  |  |  |
| Prior stroke | 1.80 | 0.20-15.84 | .60 |  |  |  |  |
| Smoking history | 2.83 | 0.33-24.44 | .35 |  |  |  |  |
| Current smoking | 5.17 | 0.60-44.69 | .14 |  |  |  |  |
| NYHA classification III or IV | 0.52 | 0.10-2.63 | .43 |  |  |  |  |
| Pro-BNP>600pg/ml | 4.48 | 0.72-27.74 | .11 |  |  |  |  |
| LVESD>40mm | 11.34 | 2.19-58.76 | .004 |  | 5.12 | 0.17-153.18 | .35 |
| LVEDD>60mm | 13.27 | 2.23-79.11 | .005 |  | 8.34 | 0.21-328.49 | .26 |
| LVEF<40% | 29.42 | 4.49-192.67 | <.001 |  | - | - | .97 |
| LVEF<50% | 4.94 | 0.87-27.99 | .07 |  | - | - | .97 |
| Anemia | 1.64 | 0.30-9.13 | .57 |  |  |  |  |
| Abnormal platelet count | - | - | .97 |  |  |  |  |
| LM stenosis | 0.53 | 0.06-4.76 | .57 |  |  |  |  |
| Three-system disease | 0.74 | 0.12-4.51 | .75 |  |  |  |  |
| **Surgical factors** |  |  |  |  |  |  |  |
| Non-elective operation | 5.35 | 0.96-29.66 | .06 |  | 4.03 | 0.57-28.57 | .16 |
| RA to LAD (non-LIMA to LAD) | - | - | .98 |  |  |  |  |
| Non-BIMA | - | - | .98 |  |  |  |  |
| Stenosis of RA targeted coronary artery<70% | 5.26 | 0.55-50.42 | .15 |  |  |  |  |
| Arterial grafts<50% | - | - | .99 |  |  |  |  |
| Non-TAR | 0.78 | 0.14-4.35 | .77 |  |  |  |  |
| Incomplete revascularization | - | - | .96 |  |  |  |  |
| On-pump | 11.80 | 1.19-116.95 | .04 |  | 11.58 | 0.60-221.71 | .10 |
| **Postoperative Medication** |  |  |  |  |  |  |  |
| CCB for 6 months | - | - | >.99 |  |  |  |  |

Table S21. Univariate and Multivariate Logistic Regression Analysis of Risk Factors for Stroke in the early period

|  | Univariate Estimates | | |  | Adjusted Estimates | | |
| --- | --- | --- | --- | --- | --- | --- | --- |
|  | OR | 95% CI | P |  | OR | 95% CI | P |
| **Preoperative factors** |  |  |  |  |  |  |  |
| Female | - | - | .98 |  |  |  |  |
| BMI ≥24 | - | - | .95 |  |  |  |  |
| BMI ≥28 | - | - | .96 |  |  |  |  |
| Aged 60 years or more | - | - | .95 |  |  |  |  |
| Aged 65 years or more | - | - | .97 |  |  |  |  |
| Aged 70 years or more | - | - | .98 |  |  |  |  |
| ACS at admission | - | - | .95 |  |  |  |  |
| MI at admission | - | - | .97 |  |  |  |  |
| Diabetes mellitus | - | - | .95 |  |  |  |  |
| Hypertension | - | - | .96 |  |  |  |  |
| COPD | 5.00 | 1.17-21.39 | .03 |  | - | - | - |
| Hyperlipemia | - | - | .96 |  |  |  |  |
| CKD | - | - | .98 |  |  |  |  |
| PVD | - | - | .98 |  |  |  |  |
| Prior MI | - | - | .95 |  |  |  |  |
| Prior stroke | - | - | .97 |  |  |  |  |
| Smoking history | - | - | .95 |  |  |  |  |
| Current smoking | - | - | .96 |  |  |  |  |
| NYHA classification III or IV | - | - | .95 |  |  |  |  |
| Pro-BNP>600pg/ml | - | - | .97 |  |  |  |  |
| LVESD>40mm | - | - | .98 |  |  |  |  |
| LVEDD>60mm | - | - | .98 |  |  |  |  |
| LVEF<40% | - | - | .99 |  |  |  |  |
| LVEF<50% | - | - | .98 |  |  |  |  |
| Anemia | - | - | .96 |  |  |  |  |
| Abnormal platelet count | - | - | .98 |  |  |  |  |
| LM stenosis | - | - | .95 |  |  |  |  |
| Three-system disease | - | - | .95 |  |  |  |  |
| **Surgical factors** |  |  |  |  |  |  |  |
| Non-elective operation | - | - | .96 |  |  |  |  |
| RA to LAD (non-LIMA to LAD) | - | - | .98 |  |  |  |  |
| Non-BIMA | - | - | .98 |  |  |  |  |
| Stenosis of RA targeted coronary artery<70% | - | - | .98 |  |  |  |  |
| Arterial grafts<50% | - | - | .99 |  |  |  |  |
| Non-TAR | - | - | .97 |  |  |  |  |
| Incomplete revascularization | - | - | .96 |  |  |  |  |
| On-pump | - | - | .99 |  |  |  |  |
| **Postoperative Medication** |  |  |  |  |  |  |  |
| CCB for 6 months | - | - | >.99 |  |  |  |  |

Table S22. Univariate and Multivariate Logistic Regression Analysis of Risk Factors for repeat revascularization in the early period

|  | Univariate Estimates | | |  | Adjusted Estimates | | |
| --- | --- | --- | --- | --- | --- | --- | --- |
|  | OR | 95% CI | P |  | OR | 95% CI | P |
| **Preoperative factors** |  |  |  |  |  |  |  |
| Female | - | - | .97 |  |  |  |  |
| BMI ≥24 | 2.69 | 0.31-23.27 | .37 |  |  |  |  |
| BMI ≥28 | 4.76 | 0.94-24.14 | .06 |  | - | - | - |
| Aged 60 years or more | 1.01 | 0.18-5.61 | .99 |  |  |  |  |
| Aged 65 years or more | - | - | .97 |  |  |  |  |
| Aged 70 years or more | - | - | .98 |  |  |  |  |
| ACS at admission | 0.46 | 0.09-2.33 | .35 |  |  |  |  |
| MI at admission | - | - | .97 |  |  |  |  |
| Diabetes mellitus | 0.33 | 0.04-2.89 | .32 |  |  |  |  |
| Hypertension | - | - | .96 |  |  |  |  |
| COPD | - | - | .98 |  |  |  |  |
| Hyperlipemia | 0.49 | 0.09-2.69 | .41 |  |  |  |  |
| CKD | - | - | .98 |  |  |  |  |
| PVD | - | - | .97 |  |  |  |  |
| Prior MI | 0.89 | 0.16-4.90 | .89 |  |  |  |  |
| Prior stroke | - | - | .97 |  |  |  |  |
| Smoking history | 1.14 | 0.21-6.32 | .88 |  |  |  |  |
| Current smoking | 2.08 | 0.38-11.49 | .40 |  |  |  |  |
| NYHA classification III or IV | 2.70 | 0.31-23.74 | .37 |  |  |  |  |
| Pro-BNP>600pg/ml | - | - | .97 |  |  |  |  |
| LVESD>40mm | 2.13 | 0.24-18.80 | .50 |  |  |  |  |
| LVEDD>60mm | 4.96 | 0.54-45.30 | .16 |  |  |  |  |
| LVEF<40% | - | - | .99 |  |  |  |  |
| LVEF<50% | 1.87 | 0.21-16.45 | .57 |  |  |  |  |
| Anemia | - | - | .96 |  |  |  |  |
| Abnormal platelet count | 1.94 | 0.22-17.05 | .55 |  |  |  |  |
| LM stenosis | 1.05 | 0.19-5.80 | .96 |  |  |  |  |
| Three-system disease | 1.00 | 0.18-5.52 | >.99 |  |  |  |  |
| **Surgical factors** |  |  |  |  |  |  |  |
| Non-elective operation | 0.50 | 0.06-4.37 | .54 |  |  |  |  |
| RA to LAD (non-LIMA to LAD) | - | - | .98 |  |  |  |  |
| Non-BIMA | - | - | .98 |  |  |  |  |
| Stenosis of RA targeted coronary artery<70% | 4.17 | 0.46-37.50 | .20 |  |  |  |  |
| Arterial grafts<50% | - | - | .99 |  |  |  |  |
| Non-TAR | 0.30 | 0.04-2.63 | .28 |  |  |  |  |
| Incomplete revascularization | 1.79 | 0.32-9.92 | .51 |  |  |  |  |
| On-pump | - | - | .99 |  |  |  |  |
| **Postoperative Medication** |  |  |  |  |  |  |  |
| CCB for 6 months | - | - | >.99 |  |  |  |  |
